# Polymerized Laminin-332 Matrix Supports Rapid and Tight Adhesion of Keratinocytes, Suppressing Cell Migration

**DOI:** 10.1371/journal.pone.0035546

**Published:** 2012-05-01

**Authors:** Yoshinobu Kariya, Hiroki Sato, Naoko Katou, Yukiko Kariya, Kaoru Miyazaki

**Affiliations:** 1 Division of Cell Biology, Kihara Institute for Biological Research, Yokohama City University, Yokohama, Japan; 2 Kihara Memorial Yokohama Foundation for the Advancement of Life Sciences, Yokohama, Japan; 3 Graduate School of Integrated Science, Yokohama City University, Yokohama, Japan; University of Pittsburgh, United States of America

## Abstract

Laminin-332 (α3ß3γ2) (Lm332) supports the stable anchoring of basal keratinocytes to the epidermal basement membrane, while it functions as a motility factor for wound healing and cancer invasion. To understand these contrasting activities of Lm332, we investigated Lm332 matrices deposited by normal human keratinocytes and other Lm332-expressing cell lines. All types of the cells efficiently deposited Lm332 on the culture plates in specific patterns. On the contrary, laminins containing laminin ß1 and/or γ1 chains, such as Lm511 and Lm311, were not deposited on the culture plates even if secreted into culture medium. The Lm332 deposition was not inhibited by function-blocking antibodies to the α3 and α6 integrins but was inhibited by sodium selenate, suggesting that sulfated glycosaminoglycans on cell surface, *e.g.* heparan sulfate proteoglycans, might be involved in the process. HEK293 cells overexpressing exogenous Lm332 (Lm332-HEK) almost exclusively deposited Lm332 on the plates. The deposited Lm332 matrix showed a mesh-like network structure as analyzed by electron microscopy, suggesting that Lm332 was highly polymerized. When biological activity was analyzed, the Lm332 matrix rather suppressed the migration of keratinocytes as compared with purified Lm332, which highly promoted the cell migration. The Lm332 matrix supported adhesion of keratinocytes much more strongly and stably than purified Lm332. Integrin α3ß1 bound to the Lm332 matrix at a three times higher level than purified Lm332. Normal keratinocytes prominently showed integrin α6ß4-containing, hemidesmosome-like structures on the Lm332 matrix but not on the purified one. These results indicate that the polymerized Lm332 matrix supports stable cell adhesion by interacting with both integrin α6ß4 and α3ß1, whereas unassembled soluble Lm332 supports cell migration.

## Introduction

The interaction of animal cells with various extracellular matrix (ECM) molecules plays critical roles in both tissue construction and regulation of cellular functions such as cell adhesion, migration, proliferation and differentiation [1], [2]. After secretion from cells, most ECM proteins are assembled into a large and complex matrix network by self-polymerization and/or interaction with other molecules [3]. Basement membrane (BM) is a thin sheet of specialized ECM, in which ECM proteins such as laminins, type IV collagen, nidogens and perlecan are assembled into a complex mesh-like membrane structure [3], [4]. It remains uncertain how each ECM molecule is assembled into the BM structure. In the BMs of various types of tissues, laminins play major roles in regulating cellular functions. Like other ECM proteins, the biological activity of laminins can be analyzed using purified proteins. However, it seems very likely that the biological activity of assembled ECM proteins differs from that of isolated proteins [5].

One of the laminin isoforms, laminin-332 (Lm332; previously known as laminin-5), which consists of laminin α3, ß3 and γ2 chains, is a major component of BMs in the skin and other stratified squamous epithelial tissues [6], and associates with integrin α6ß4 to form the stable adhesion structure hemidesmosome [7], [8]. Therefore, genetic mutations of Lm332 subunits cause a severe and lethal skin blistering disease, Herlitz’s junctional epidermolysis bullosa [9], [10]. *In vitro,* Lm332 promotes cellular adhesion, motility and scattering [11]–[13]. These activities are mainly mediated through the interaction of the C-terminal laminin globular (LG) domain of the α3 chain, especially the LG3 domain with integrins α3ß1, α6ß1 and α6ß4 [14], [15]. Lm332 has unique activity that even in a soluble form, it induces cell migration and scattering via PKC, phosphatidylinositol 3-kinase (PI3K) and ERK activation by binding to integrins α3ß1 and α6ß1 on apical cell surface [16]. *In vivo*, expression of Lm332 is induced at the wounded edge of epidermis and at the leading edge of invading carcinomas. Therefore, Lm332 is thought to contribute to cell migration in wound healing [17], [18] and tumor invasion [19], [20]. Indeed, keratinocytes deficient in Lm332 expression show defects in their cell migration [18], [21]. These facts suggest a crucial role of Lm332 in the migration of normal keratinocytes as well as invading cancer cells. In contrast, there is a report showing that Lm332 inhibits keratinocyte migration *in vitro* [22]. Thus, Lm332 seems to exhibit two opposite activities, stable adhesion and cell motility both *in vivo* and *in vitro*.

Proteolytic processing of Lm332 may be at least in part responsible for the contrasting activity of Lm332. There are reports showing that the cleavage of the laminin α3 chain from the precursor (190 kDa) to the mature (160 kDa) form decreases the cell migration activity of Lm332 [23]. However, our previous study with recombinant Lm332 showed that both cell adhesion and motility activities of Lm332 are enhanced when the α3 chain is processed to the mature form [24]. On the other hand, the proteolytic cleavage of the laminin γ2 chain seems to be more important for the Lm332-mediated cell migration than that of the α3 chain. Previous studies showed that the cleavage of the γ2 chain from the precursor (150 kDa) to the mature (105 kDa) form significantly increases the cell migration activity of Lm332 [25], [26]. However, because the difference in the cell motility activity between the two forms of Lm332 is not very striking [27], it is difficult to consider that only proteolytic processing could be responsible for the differential cell motility activity of Lm332 [28].

Many previous studies have shown that normal keartinocytes deposit Lm332 onto the surface of culture plates [29]. This Lm332-containing matrix may have a different biological activity from that of purified Lm332. In addition, it is important to investigate how Lm332 is deposited and assembled into the matrix after secretion. We previously established HEK293 cell lines overexpressing recombinant Lm332 (Lm332-HEK) [30] or laminin-3B32 (Lm3B32-HEK) [31]. They secrete and deposit Lm332 or Lm3B32 at a high level in culture. Using Lm332-HEK and other Lm332-expressing cell lines, we here investigated the assembly of Lm332 into matrix and its biological activity, comparing the activities of the deposited Lm332 matrix and purified Lm332 protein.

## Results

### Deposition of Lm332 Matrix by Normal and Cancer Cells

**Figure 1:**
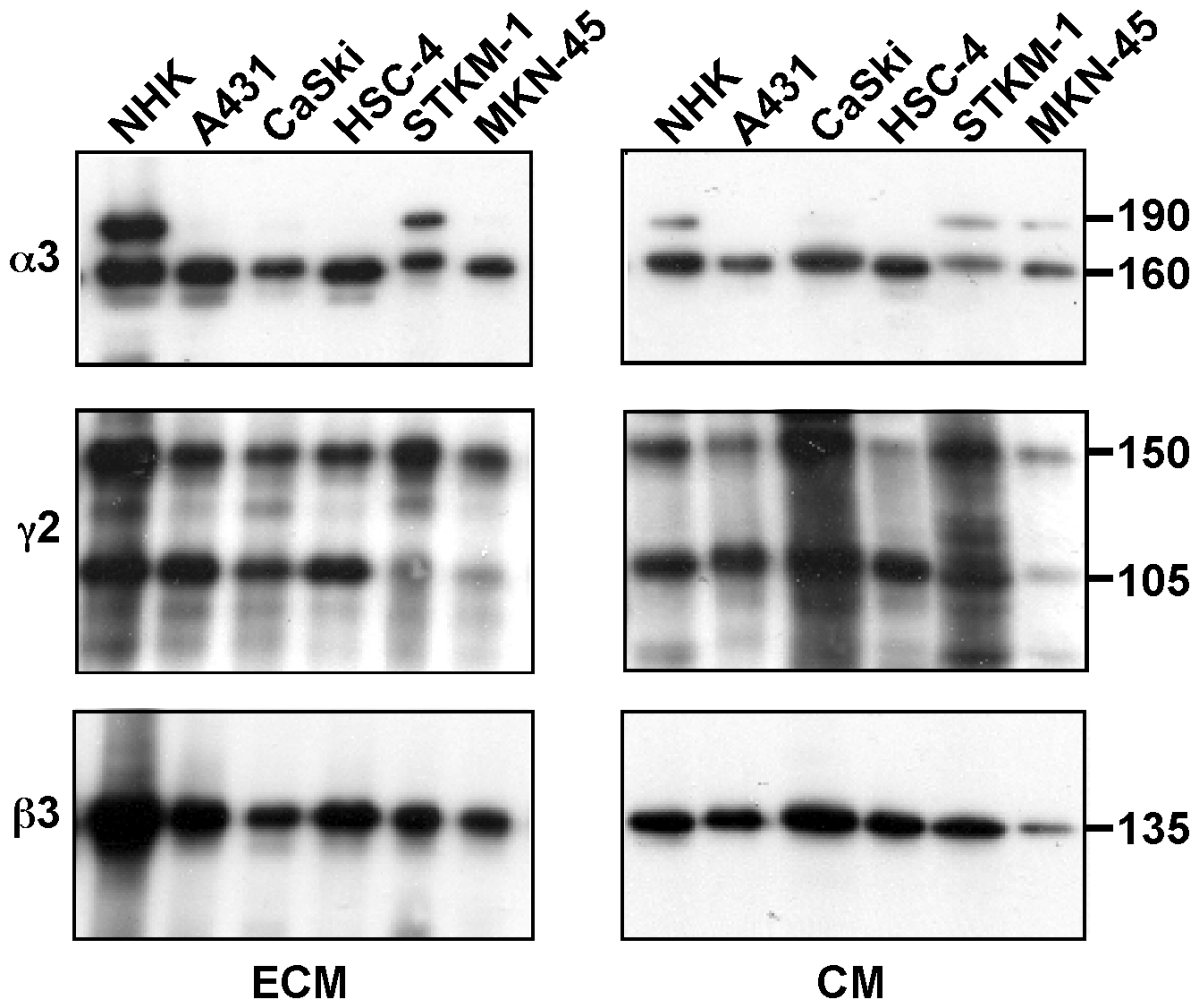
Deposition of Lm332 on culture plates by normal keratinocytes (NHK) and five cancer cell lines (A431, CaSki, HSC-4, STKM-1 and MKN-45). Cells were suspended in serum-free medium, inoculated at a density of 5×10^6^ cells per 90-mm dish, and incubated for 2 days. The resulting ECM and CM were prepared from each culture. To prepare ECM, cells were removed from the dishes by treating them with 10 mM EDTA and briefly with 20 mM NH_4_ OH and then washing with PBS. The ECMs on the dishes were extracted with the SDS sample buffer. A twentieth part of the CM and a thirtieth part of the ECM were subjected to immunoblotting with the antibodies to the laminin α3, γ2 and ß3 chains under reducing conditions. Bars indicate the position and size of the laminin chains. Other experimental conditions are described in “Materials and Methods”.

Lm332 is expressed by various kinds of normal epithelial cells and cancer cells [11], [32]. The C-terminal LG4-5 domain of the α3 chain and the N-terminal short arm of the γ2 chain, both of which are liberated by proteolytic processing, regulate the matrix assembly and activity of Lm332 [33], [34]. To characterize the Lm332-containing matrices, we first analyzed Lm332 secreted and deposited by primary normal human epidermal keratinocytes (NHK), three squamous cell carcinoma cell lines (A431, CaSki, and HSC-4) and two gastric adenocarcinoma cell lines (STKM-1 and MKN-45) by Western blotting. The relative amount of the deposited Lm332 in the ECM to the soluble one in the conditioned medium (CM) was highest in NHK cells, but all cancer cell lines deposited considerable amounts of Lm332 on the plastic surface (Figure 1, left column). In the deposited ECM, the 190-kDa precursor α3 chain, which contains the LG4-5 domain, was detected only in the cultures of NHK and STKM-1 cells. All ECMs (Figure 1, left column) and CM (Figure 1, right column) contained both the 150-kDa precursor and 105-kDa processed γ2 chains, but the relative amount of the processed form to the precursor was higher in the CM than the ECMs. A nearly single band of ß3 chain was detected in all samples, indicating that this chain is relatively resistant to the proteolytic cleavage.

**Figure 2:**
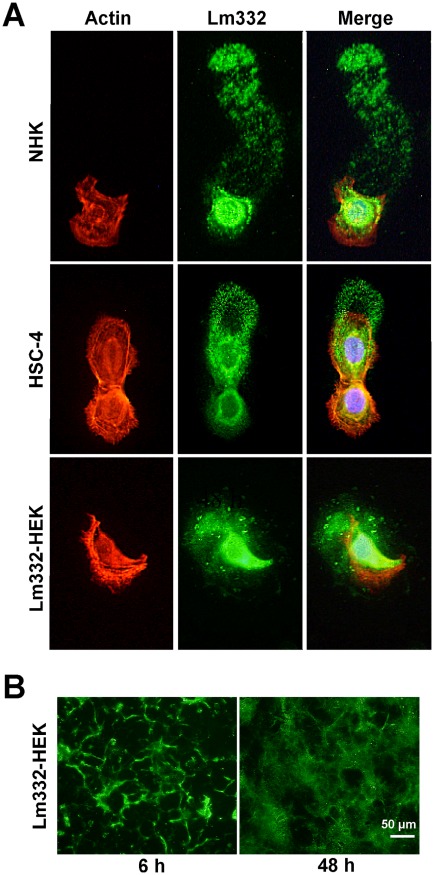
Immunofluorescent staining of Lm332 deposited by three cell lines. NHK (A, top), HSC-4 cells (A, center) and Lm332-HEK cells (A. bottom) were suspended in serum-free medium, inoculated at a cell density of 2×10^3^ cells/well on collagen-coated 8-well chamber slides and incubated for 6 h. The cultures were stained for F-actin with rhodamine phalloidin (left panels) and for Lm332 with the anti-α3 chain BG5 antibody and followed by a FITC-labeled secondary antibody (center panels), as described in “Materials and Methods”. Right panels are merged images. In (B), Lm332-HEK cells were inoculated at a high cell density (1×10^5^ cells/well), incubated for 6 h (left panel) or 48 h (right panel), and stained for Lm332 as above.

To see how Lm332 is deposited, the Lm332 deposition was visualized by immunofluorescent staining with the anti-α3 chain antibody BG5 in sparse cultures (Figure 2A). In agreement with a previous study [35], migrating NHK deposited many Lm332 signals on their trails (Figure 2A, upper panels, Lm332). In a stationary HSC-4 cell, Lm332 was most densely observed in a perinuclear circle area and co-localized only with perinuclear actin filaments (a lower cell in Figure 2A, middle panels, Merged). A slowly migrating cell deposited these Lm332 proteins as spike-like or arrowhead-like spots in a semicircle area. We have previously established HEK293 cell lines which overexpress recombinant Lm332 (Lm332-HEK) [30]. Slowly migrating Lm332-HEK cells produced and left different sizes of Lm332 spots uniformly behind the cells (Figure 2A, lower panels, Lm332). However, when Lm332-HEK cells were plated at a high density, they initially deposited Lm332 in peripheral regions of individual cells (Figure 2B, left panel), but further deposition of Lm332 covered whole surface of the culture plates with cotton-like fibers (Figure 2B, right panel). We also examined the patterns of Lm332 matrices deposited by confluent cultures of NHK and cancer cell lines (Figure S1). NHK, A431 and HSC-4 produced a cloud-like or rosette-like pattern of Lm332 deposition, where small ring structures were visible especially in the matrices of NHK and A431 (Figure S1). Compared to these matrices, two gastric adenocarcinoma cell lines (STKM-1 and MKN-45) produced relatively homogeneous Lm332 matrix with spiny or fibrous structures (Figure S1). These results suggest that Lm332 is initially deposited in perinuclear or more peripheral regions, and the differences in the Lm332 deposition patterns may largely depend on the motility and cytoskeletal structure of Lm332-expressing cells. Similar Lm332 patterns were obtained when the Lm332 matrices were immunostained with antibodies recognizing the laminin α3, ß3 and γ2 chains (Figure S2).

**Figure 3:**
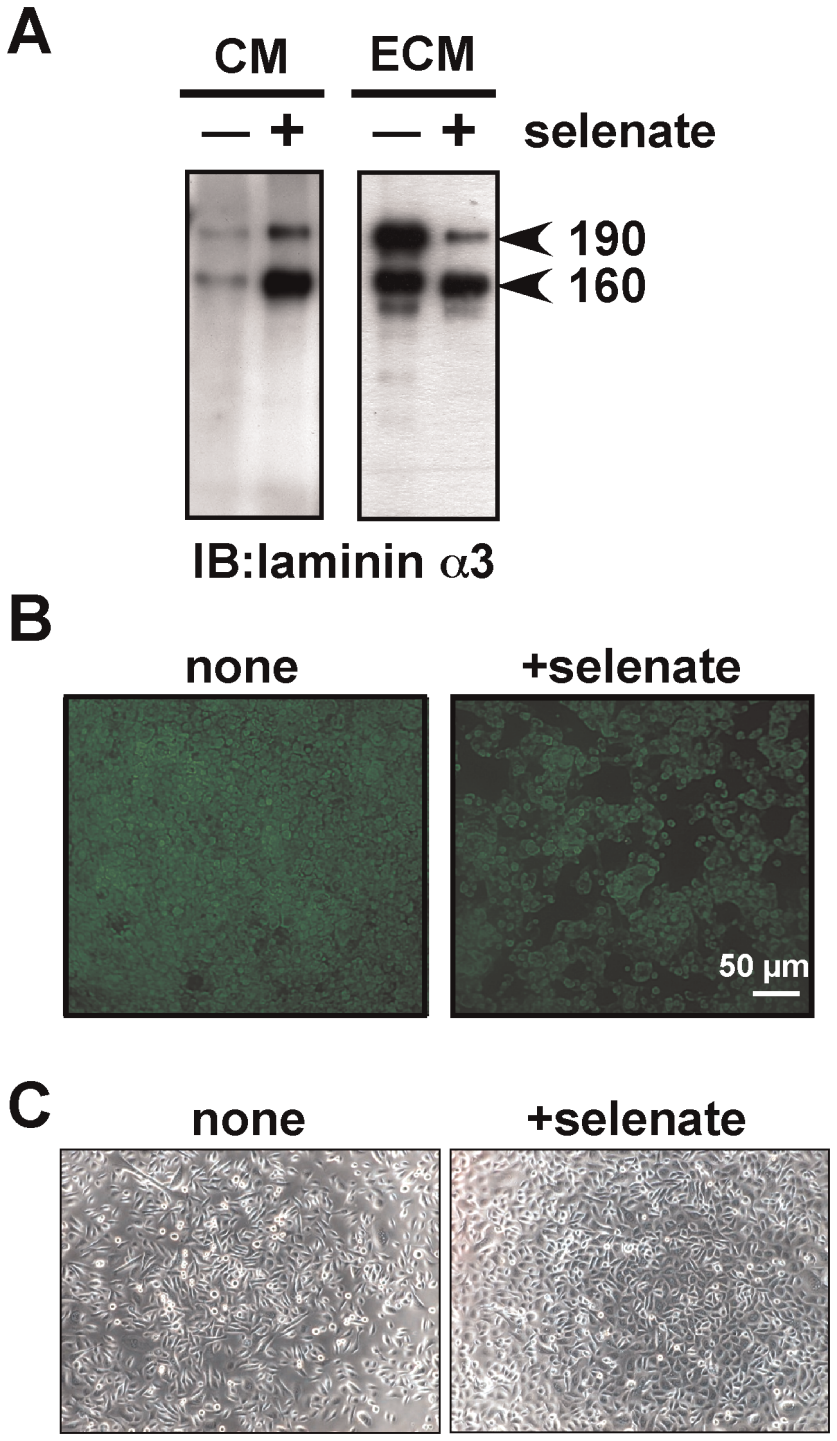
Effect of sodium selenate on Lm332 deposition by NHK cells. (A) Immunoblotting analysis. NHK cells were inoculated in serum-free medium at a density of 4×10^5^ cells per 35-mm dish, incubated overnight, and treated with (+) or without (−) 0.1 mM sodium selenate (Sigma) at 37°C for 24 h. After the incubation, the ECM and CM were prepared from each culture and analyzed for the laminin α3 chain by immunoblotting as described in Figure 1. (B) The ECMs from the control (none) and selenate-treated cultures (+selenate) were subjected to immunofluorescence staining with the anti-laminin α3 chain antibody BG5. (C) Phase-contrast micrographs of control and selenate-treated cultures. Other experimental conditions are described in “Materials and Methods”.

It has been reported that the Lm332 deposition or its assembly to ECM is mediated by cell surface molecules [35]–[37]. We examined the possible role of integrins in the Lm332 deposition. Although a mixture of function-blocking anti-integrin-α3 and anti-integrin-α6 antibodies completely blocked cell adhesion to Lm332-coated plates, it did not affect the Lm332 deposition on collagen-coated plates (Figure S3). On the other hand, sodium selenate, an inhibitor for the sulfation of glycosaminoglycans, inhibited the Lm332 deposition of NHK cells onto culture plates as analyzed by immunoblotting (Figure 3A) and immunocytochemistry for the lamininα3 chain (Figure 3B), under nontoxic conditions (Figure 3C). These results strongly suggest that the Lm332 deposition is mainly mediated by cell surface sulfated glycosaminoglycans, *e.g.* heparan sulfate proteoglycans like syndecans, but not by integrins.

### Characterization of Lm332 Matrix Deposited by Lm332-HEK Cells

**Figure 4:**
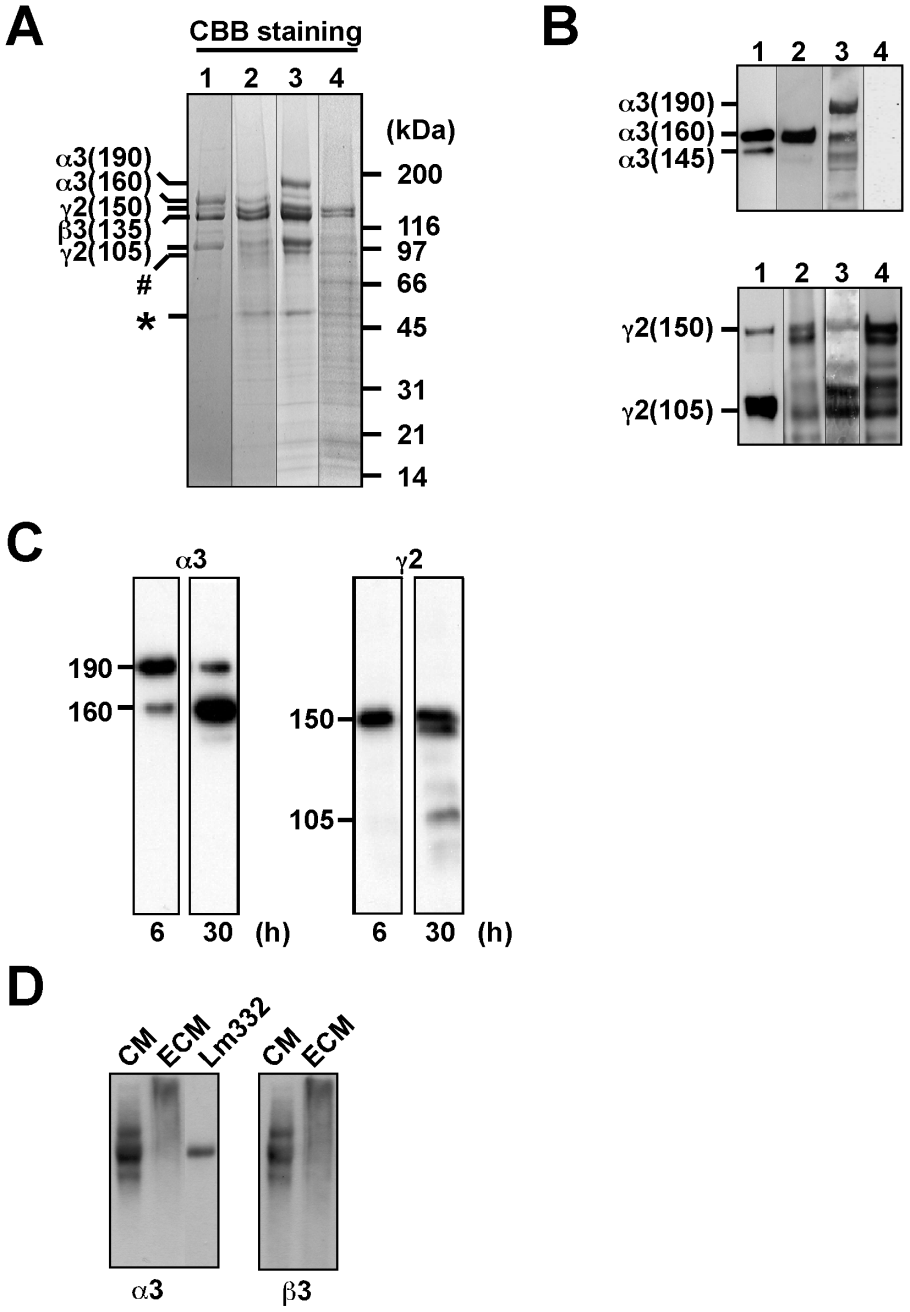
SDS-PAGE analyses of Lm332-ECM deposited by Lm332-HEK cells. Confluent cultures of Lm332-HEK (lane 2), α3AA-Lm332-HEK (lane 3) and ß3γ2-HEK (lane 4) were incubated in the serum-containing growth medium for 4 days, and the resultant ECMs were prepared and applied to SDS-PAGE under reducing conditions, followed by CBB staining (A) or immunoblotting with anti-α3 (B, upper panel) and -γ2 (B, lower panel) chain antibodies. Purified Lm332 was run as a standard on lane 1. Bars on the left indicate major protein bands with their approximate molecular sizes in kDa. The two minor bands (# and *) in (A) were identified as laminin γ2 fragments by NH_2_-terminal amino acid sequencing. Other experimental conditions are described in “Materials and Methods”. (C) To see the proteolytic processing of deposited Lm332, Lm332-HEK cells were incubated for 6 h and 30 h, and the deposited Lm332 was prepared and applied to SDS-PAGE under reducing conditions, followed by immunoblotting for the α3 chain (left panels) and the γ2 chain (right panels). (D) CM (lane 1) and ECM (lane 2) were prepared from the confluent culture of Lm332-HEK cells after incubation in serum-free medium for 2 days and analyzed by immunoblotting under non-reducing conditions with anti-α3 (left panel) and -ß3 (right panel) chain antibodies. Lane 3, purified Lm332 with processed γ2 chain (400 kDa). Note that Lm332 in the ECM remains on the gel top as a broad band (lane 2).

To characterize the Lm332-containing matrix biochemically and biologically, we used Lm332-HEK and related HEK293 cell lines, as well as purified recombinant Lm332 protein. ECMs were prepared from the cultures of Lm332-HEK [30], α3AALm332-HEK, which overexpresses an α3 chain-mutated Lm332 resistant to proteolytic processing [24], and ß3γ2-HEK, which had been transfected only with the laminin ß3 and γ2 chain cDNAs [30]. The ECMs and purified Lm332 were analyzed by SDS-PAGE and subsequent Coomassie Brilliant Blue (CBB) staining or immunoblotting. The CBB staining showed that Lm332-HEK (Figure 4A, lane 2) and α3AALm332-HEK (Figure 4A, lane 3) cell lines almost exclusively deposited the three chains of Lm332 and their proteolytic fragments. We identified two proteolytic fragments of laminin γ2 chain at approximately 90-kDa (#) and 50-kDa (*). NH_2_-terminal amino acid sequencing revealed that the 90-kDa protein had the same NH_2_-terminal sequence as the mature 105-kDa γ2 chain, while the 50-kDa protein was the NH_2_-terminal fragment separated from the 105-kDa γ2 chain. These fragments were also present in the CM of Lm332-HEK cells (data not shown). Furthermore, this analysis showed that ß3γ2-HEK cells secreted and deposited the ß3 and γ2 chains (Figure 4A, lane 4). As shown by immunoblotting (Figure 4B, upper panel) as well as the CBB staining (Figure 4A), α3AALm332-HEK cells deposited the 190-kDa precursor (or unprocessed) α3 chain as a major component, whereas this was never or scarcely detected in the purified Lm332 and the ECM of Lm332-HEK (named Lm332-ECM), both of which contained the 160-kDa mature (or processed) α3 chain as a major component. Immunoblotting for the γ2 chain showed that the Lm332-ECM contained the 150-kDa γ2 chain more than the 105-kDa processed γ2 chain, but vice versa in the ECM of α3AALm332-HEK and the purified Lm332 (Figure 4B, lower panel).

When the ECM was prepared from Lm332-HEK cultures 6 and 30 h after inoculation, the 190-kDa unprocessed α3 chain was found as a major band at 6 h but it was mostly converted to the 160-kDa processed form for 30 h (Figure 4C). Similarly, the 150-kDa precursor γ2 chain was found as a single band at 6 h, but it was partially converted to the 140- and 105-kDa processed forms for 30 h. These results suggest that the proteolytic cleavage of these chains mainly occurs after Lm332 is deposited and the processing of the α3 chain is much faster than that of the γ2 chain.

When Lm332-ECM prepared from a 2-days culture was separated by SDS-PAGE under non-reducing conditions, Lm332 heterotrimer could not enter the separating gel (Figure 4D). The same was true even in the Lm332-ECM from a 6-h culture (data not shown). Since Lm332 in the ECM was separated into its three subunits by reducing SDS-PAGE (Figure 4A), Lm332 was supposed to be polymerized by cross-linkage with disulfide bonds.

The density of Lm332 in the ECM deposited by Lm332-HEK cells was analyzed by Enzyme-linked immunosorbent assay (ELISA) with monoclonal antibodies against the laminin α3 and γ2 chains. The Lm332 concentration on the plate was equivalent to that obtained by coating purified Lm332 at a concentration of 0.56 µg/ml and 0.61 µg/ml as analyzed for the α3 and γ2 chains, respectively (Figure S4, A and B). SDS-PAGE analysis verified that this level of Lm332 was indeed present in Lm332-ECM (Figure S4, C). The ELISA also showed that the concentration of the γ2 chain in the ECM deposited by ß3γ2-HEK cells was almost the same as that in the Lm332-ECM.

It is likely that ECM proteins other than Lm332 are also assembled into Lm332-ECM. We have previously reported that HEK293 cells secrete laminin-511 (Lm511) into the culture medium [38]. In immunoblotting analysis, the laminin α5 chain was detected at lower molecular sizes than the authentic α5 chain of recombinant Lm511 in the CM of Lm322-HEK cells, but it was undetectable in the ECM (Figure S5A). Similarly, the laminin ß1 and γ1 chains were detected only in the CM. As shown in Figure S1, two gastric carcinoma cell lines showed characteristic patterns of the Lm332 matrix. Therefore, we also analyzed other laminins in the CM and ECM of MKN45 cell line. Non-reducing immunoblotting analysis showed that the CM of MKN45 gastric carcinoma cells contained both Lm332 and laminin-311 (Lm311) at comparable levels, but neither the laminin ß1 nor γ1 chain was detected in the ECM (Figure S6). These results suggest that in contrast to Lm332, laminins containing laminin ß1 and/or γ1 chains, such as Lm511 and Lm311, are hardly deposited on the culture plates. Furthermore, we analyzed fibronectin, nidogen-1, perlecan, type IV collagen and type VII collagen in the ECM and CM of Lm332-HEK cells. Human fibronectin and nidogen-1 were detected in the CM but they were absent in the ECM (Figure S5, B and C, respectively). Perlecan, type IV collagen and type VII collagen were undetectable in both ECM and CM of Lm332-HEK cells (data not shown).

**Figure 5:**
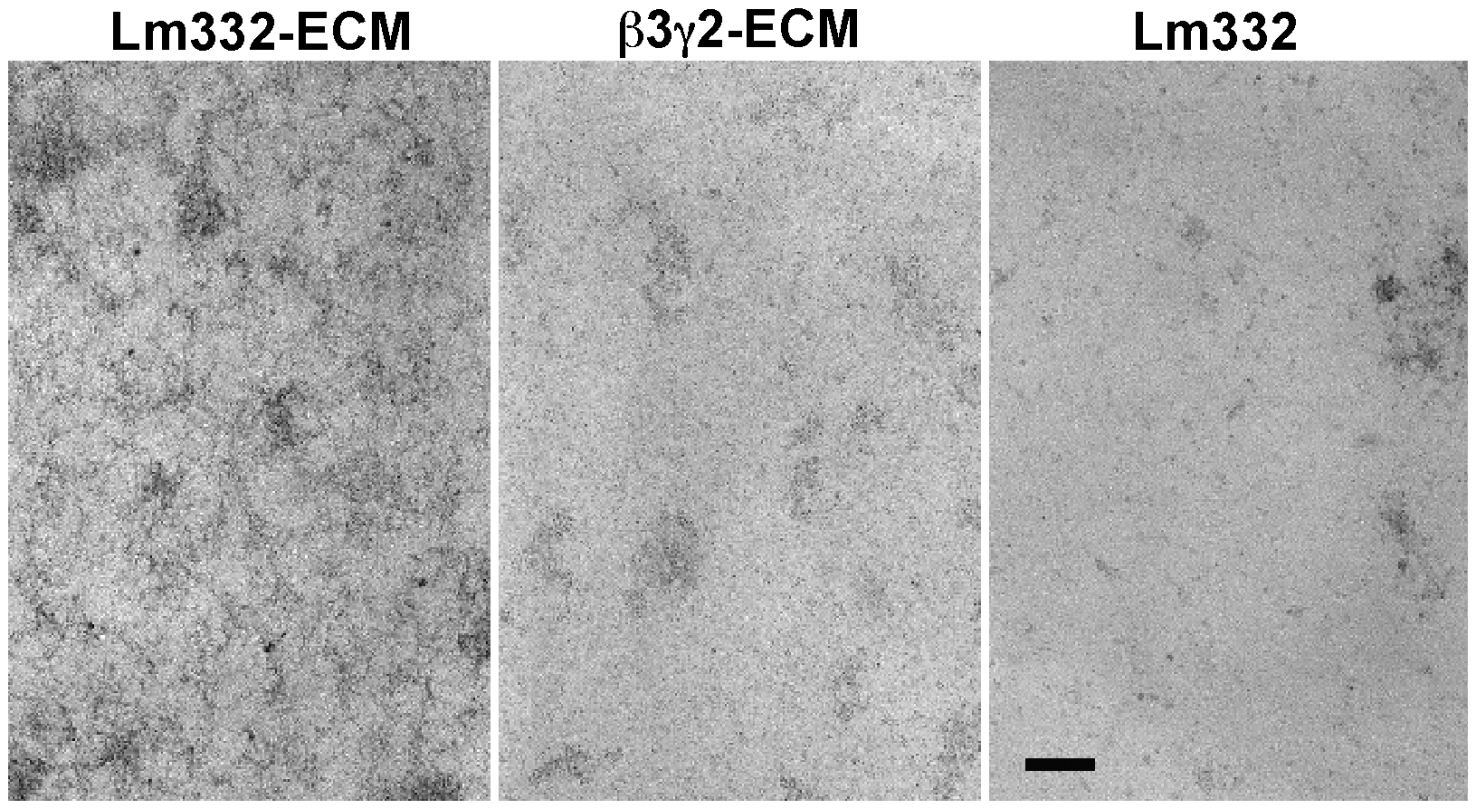
Fine structures of Lm332-ECM (left), ß3γ2-ECM (center) and purified Lm332 (right) were analyzed by TEM at a magnification of × 50,000. Bars indicate 100 nm.

Fine structure of Lm332-ECM was also analyzed by transmission electron microscopy (TEM). The TEM image of Lm332-ECM showed a mesh-like, molecular network structure covering whole surface of the glass plate, in which high density areas were distributed (Figure 5). This suggests that Lm332 may be polymerized on the basal surface of cell membrane and transferred onto the culture substrate. Such a mesh-like structure was not found in the ß3γ2-ECM, though high density areas, possibly due to protein aggregates, were distributed on the plates. Lm332-coated plates showed some protein aggregates without any clear structure, suggesting that Lm332 molecules mostly detached from the plate during the staining procedure.

### Migration of Keratinocytes on Lm332-ECM

**Figure 6:**
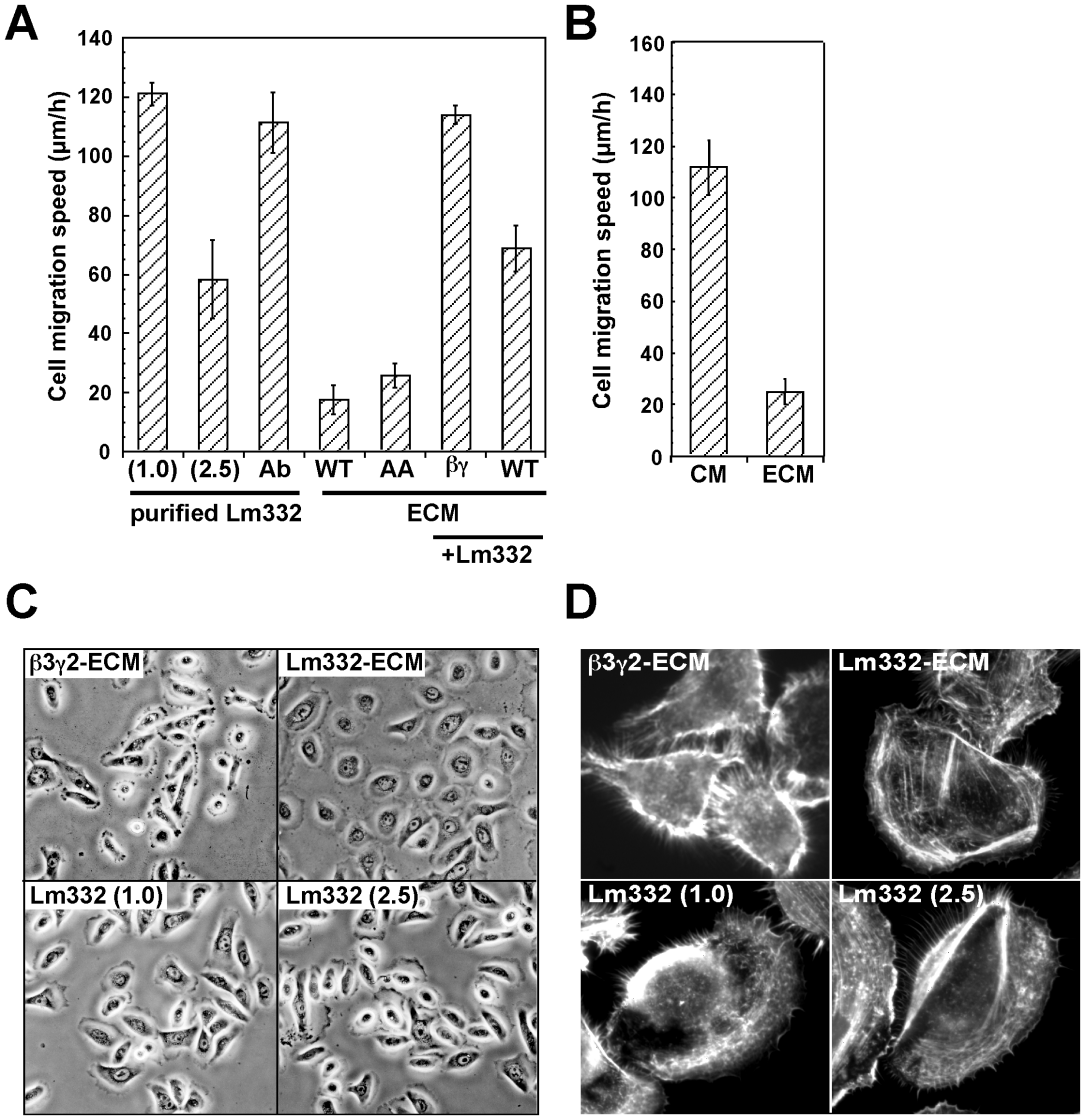
Migration and morphology of NHK cells on purified Lm332 and Lm332-ECM substrates. (A) NHK cells were inoculated on Lm332-coated plates (left three columns) or deposited ECMs (right four columns), which were prepared as described below. After 1.5 h incubation, cell migration was monitored by video microscopy for 5.5 h. Purified Lm332 was coated at a concentration of 1.0 or 2.5 µg/ml on a non-treated plate, or at 1.0 µg/ml on a plate pre-coated with the anti-laminin α3 chain antibody LSαc3 (Ab). ECM substrates were prepared from Lm332-HEK (WT), α3AA-Lm332-HEK (AA), and ß3γ2-HEK (ßγ) cultures. In the right two columns, ß3γ2-ECM and Lm332-ECM were further coated with 1.0 µg/ml purified Lm332 (+Lm332) and then used for the migration assay. On ß3γ2-ECM alone or a non-treated plate, NHK cells never attached and migrated during the initial 7 h (data not shown). Each bar represents the mean ± S.D. of the migration speeds of 8 cells in each assay. These results were essentially reproduced in three independent experiments. Other experimental conditions are described in “Materials and Methods”. (B) CM and deposited ECM (ECM) were prepared from confluent cultures of NHK cells. The CM was coated on a 24-well plate. Migration of NHK cells on these substrates was analyzed as above. (C) NHK cells were incubated for 3 h on ß3γ2-ECM, Lm332-ECM and plates coated with 1.0 or 2.5 µg/ml purified Lm332, and cell morphology was observed under a phase-contrast microscope. Original magnification, ×300. (D) In the same cultures as (C), actin cytoskeleton was visualized with FITC-phalloidin. Numerical values in parentheses indicate the concentration (µg/ml) of Lm332 coated.

To show the biological activity of Lm332-ECM, we first examined effects of Lm332-ECM and purified Lm332 on migration of NHK cells. In these assays, the cell density and incubation length were minimized to neglect the effect of endogenously secreted or deposited Lm332. When the cells were plated onto culture plates pre-coated with 1.0 µg/ml Lm332, they actively and directionally migrated on the substrate (Figures 6A and S7). When the Lm332 concentration was increased to 2.5 µg/ml, the cell migration was reduced to a half level. To obtain regular orientation of coated Lm332 molecules, we first coated a monoclonal antibody (LSαc3) that recognizes an NH_2_-terminal sequence (domain IIIa) of the α3 chain onto a plate and then bound Lm332 to the antibody-coated plate, thus allowing the integrin-binding domain LG1-3 of the α3 chain to face the cell surface receptors. This antibody-mediated Lm332-coated plate supported the rapid migration of NHK cells (Figure 6A, Ab). In contrast, NHK cells poorly migrated on both Lm332-ECM and α3AALm332-ECM (Figure 6A, WT and AA; Figure S8), regardless of the differences in the proteolytic processing of the α3 and γ2 chains as shown in Figure 4B. We could not measure the cell migration speed on ß3γ2-ECM, because NHK cells could not fully adhere to the substrate for initial 1.5 h. However, when purified Lm332 was coated on Lm332-ECM (Figure 6A, WT+Lm332) and ß3γ2-ECM (Figure 6A, ßγ+Lm332), the cell migration was significantly enhanced. These results demonstrate that coated Lm332 potently promotes migration of NHK cells, whereas Lm332-ECM rather suppresses it. The migration-suppressive activity of Lm332-ECM seemed to be independent on the orientation of Lm332 or the proteolytic processing of the α3 and γ2 chains.

As described above (Figure S1), the patterns of Lm332 deposition differed depending on cell types. To confirm the suppressive effect of Lm332-ECM on cell motility, we also examined effects of the Lm332-containing ECM and the CM obtained from the culture of NHK cells. The culture plate pre-coated with the CM, which contained a high level of Lm332 as shown in Figure 1, strongly promoted the migration of NHK cells (Figure 6B). In contrast, the Lm332-containing ECM showed the suppressive effect on the cell migration.

Cell migration requires morphological changes associated with dynamic actin cytoskeleton reorganization. Therefore, cell morphology and actin cytoskeleton were examined for NHK cells on purified Lm332, Lm332-ECM and ß3γ2-ECM. On ß3γ2-ECM, NHK cells attached but poorly spread extending filopodia-like protrusion (Figure 6C). In contrast, NHK cells efficiently attached to Lm332-ECM and spread well showing disc-like, very flat morphology. The cells on Lm332-coated plates showed refractive morphology with typical lamellipodia at the leading edge. Such polarized cells were rarely found in the cells on Lm332-ECM or ß3γ2-ECM. There was little difference in cell morphology between the Lm332 concentrations of 1.0 µg/ml and 2.5 µg/ml.

Morphological characteristics of NHK cells on the different substrates were reproduced by visualization of actin cytoskeleton with rhodamine-phalloidin (Figure 6D). NHK cells plated on purified Lm332, regardless of its coating concentration, exhibited large lamellipodia toward the moving direction and F-actin accumulation and many retraction fibers at the rear. In contrast, the cells spread on Lm332-ECM exhibited cortical actin accumulation around the cell body and some stress fibers. The cells on ß3γ2-ECM were characteristic in robust cortical actin bundles and radially extended actin filaments around cells. When NHK cells were placed on their own ECM, they showed similar morphological characteristics to the cells on Lm332-ECM. These morphological and cytoskeletal characteristics of NHK cells on the different substrates appear to reflect their motile property.

### Distinct Cell Adhesion Activity Between Lm332-ECM and Purified Lm332

**Figure 7:**
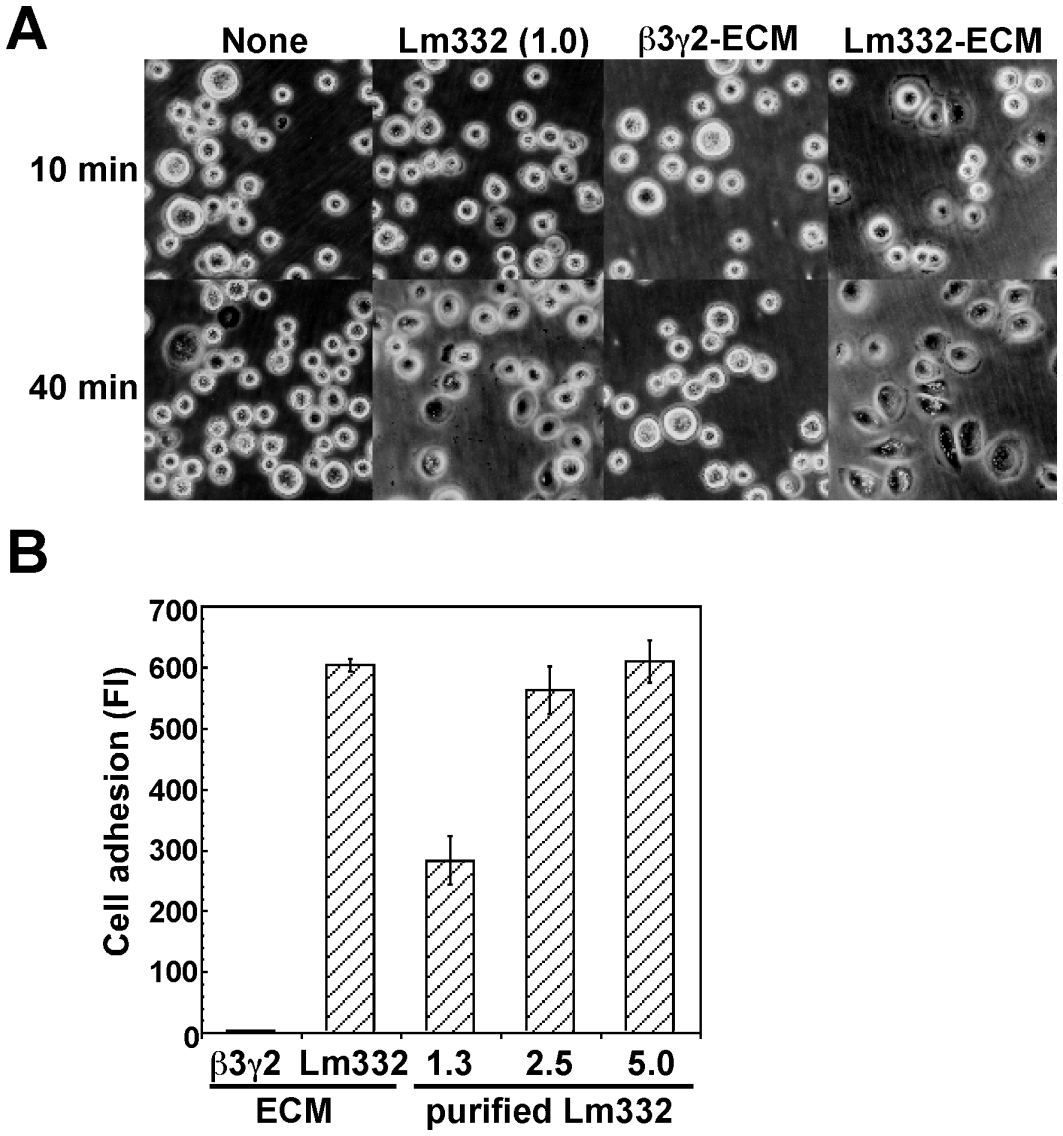
Cell adhesion activity of Lm332-ECM and purified Lm332 toward NHK cells. ECMs from ß3γ2-HEK and Lm332-HEK cultures and Lm332-coated plates were prepared as described in Figure 4. (A) Phase-contrast microscopic images of NHK cells after 10 min incubation. Original magnification, ×300. Lm332 protein was coated at 1.0 µg/ml on the plate. (B) NHK cells suspended in KGM growth medium were inoculated into each well and then incubated at 37°C for 10 min. After the incubation, non-adherent cells were removed, and adherent cells were quantified. Numerical values under three right columns indicate the concentration at µg/ml of coated Lm332 protein. Each bar represents the mean ± S.D. of the fluorescent intensity (FI) for adherent cells in triplicate assays. The data shown are representative of at least three independent experiments performed.

The results shown above suggested that the differential cell motility between purified Lm332 and Lm332-ECM might depend on difference in their cell-adhesive activity. This possibility was tested by incubating NHK cells at 37°C for 10 min or 40 min on each substrate (Figure 7A). NHK cells could not attach to ß3γ2-ECM under these conditions and even after incubation for 1.5 h. In contrast, NHK cells on Lm332-ECM attached and in parts spread at 10 min and they mostly well spread at 40 min. On a plate coated with 1.0 µg/ml Lm332, NHK cells more slowly attached and a majority of them started to spread at 40 min. Such observation was confirmed by quantitative analysis (Figure 7B). Lm332-coated plates promoted cell attachment in a dose-dependent manner. The cell attachment reached the same level at 5.0 µg/ml as that to Lm332-ECM.

**Figure 8:**
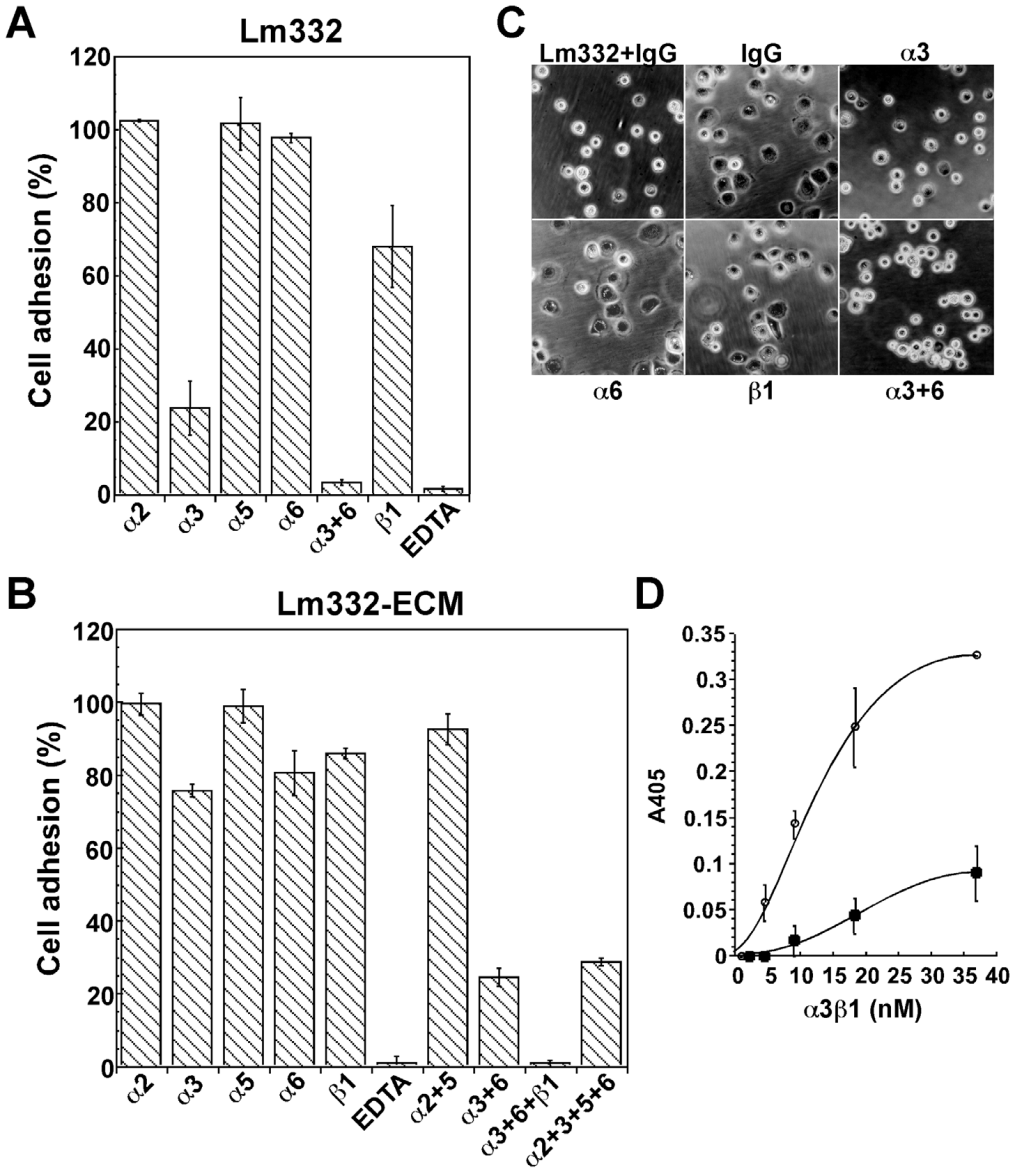
Interaction of purified Lm332 or Lm332-ECM with integrins. (A and B) To identify integrins responsible for cell adhesion, NHK cells suspended in the KGM medium were pretreated with the indicated integrin antibodies (2 µg/ml IgG) or EDTA for 30 min at room temperature and plated on the plates with purified Lm332 (A) or Lm332-ECM (B). After 20 min incubation, adherent cells were quantified. The relative number of adherent cells in the presence of control mouse or rat IgG was taken as 100%. Each bar represents the mean ± S.D. of the fluorescent intensity (FI) for adherent cells in triplicate assays. (C) Morphology of NHK cells was examined after incubation on Lm332-ECM in the presence of control mouse IgG, or the indicated anti-integrin neutral antibodies for 20 min. In the upper left culture (Lm332+IgG), NHK cells were incubated in a well precoated with 1.0 µg/ml purified Lm332 in the presence of the control IgG. Original magnification, ×300. The images shown are representative of at least three independent experiments performed. (D) Binding affinity of integrin α3ß1 to purified Lm332 and Lm332-ECM was determined by ELISA. Varied concentrations of purified integrin α3ß1 was allowed to bind to the plates coated with 1 µg/ml purified Lm332 (closed squares) or deposited with Lm332-ECM (open circles) in the presence of 1 mM MnCl_2_. Bound integrin α3ß1 was quantified by ELISA using an anti-integrin α3ß1 polyclonal antibody. The amounts of integrin α3ß1 bound in the presence of 10 mM EDTA was taken as nonspecific binding and subtracted as the background. The results shown are the means of duplicate assays.

To examine which receptors mediate the strong adhesive activity of Lm332-ECM, inhibitory effects of various function-blocking anti-integrin antibodies and EDTA were analyzed toward NHK cells (Figure 8). In agreement with previous reports (8–10), the cell attachment to purified Lm332 was effectively blocked by an anti-α3 integrin and weakly by an anti-ß1 integrin antibody, but anti-α2, -α5 and -α6 antibodies did not have such inhibitory effect (Figure 8A). A combination of anti-α3 integrin and anti-α6 integrin antibodies, as well as EDTA alone, completely blocked the cell attachment. On the other hand, the attachment of NHK cells to Lm332-ECM was scarcely blocked by any of anti-α3, -α6 and -ß1 antibodies (Figure 8B). However, a combination of anti-α3 and -α6 antibodies blocked the cell attachment to about 25%. The addition of anti-ß1 integrin antibody, but not anti-α2 or -α5 antibody, to the mixture of anti-α3 and -α6 antibodies completely blocked the cell attachment. When morphological effect was examined, the spreading of NHK cells on the Lm332-ECM was blocked partially by the anti-α3 integrin antibody and almost completely by the combination of anti-α3 and -α6 antibodies (Figure 8C). Neither anti-α6 nor anti-ß1 integrin antibody showed significant inhibition of cell spreading. ß4 integrin is known to be expressed as α6ß4, rather than α6ß1, integrin in keratinocytes [39]. Therefore, these results suggest that although NHK cells preferentially utilize integrin α3ß1 to attach to purified Lm332, integrin α6ß4 also contributes to the cell attachment to some extent. In the case of the cell attachment to Lm332-ECM, NHK cells seemed to utilize both integrins α3ß1 and α6ß4.

The results shown above suggest that the binding affinity of integrins α3ß1 and α6ß4 for Lm332-ECM may be higher than that for purified Lm332. To test this possibility, we analyzed the binding affinity of integrin α3ß1 to Lm332-ECM and purified Lm332 (Figure 8D). When purified integrin α3ß1 was added at varied concentrations into wells deposited with Lm332-ECM or those pre-coated with 1 µg/ml purified Lm332 in the presence of Mn^2+^, the integrin bound to the former at a much higher level than the latter. When integrin α3ß1 was added at 37 nM, the amount of integrin bound to Lm332-ECM was about 3.6-times higher than that to the coated Lm332 even though the actual concentration of Lm332 was higher in the latter wells (also see Figure S4).

**Figure 9:**
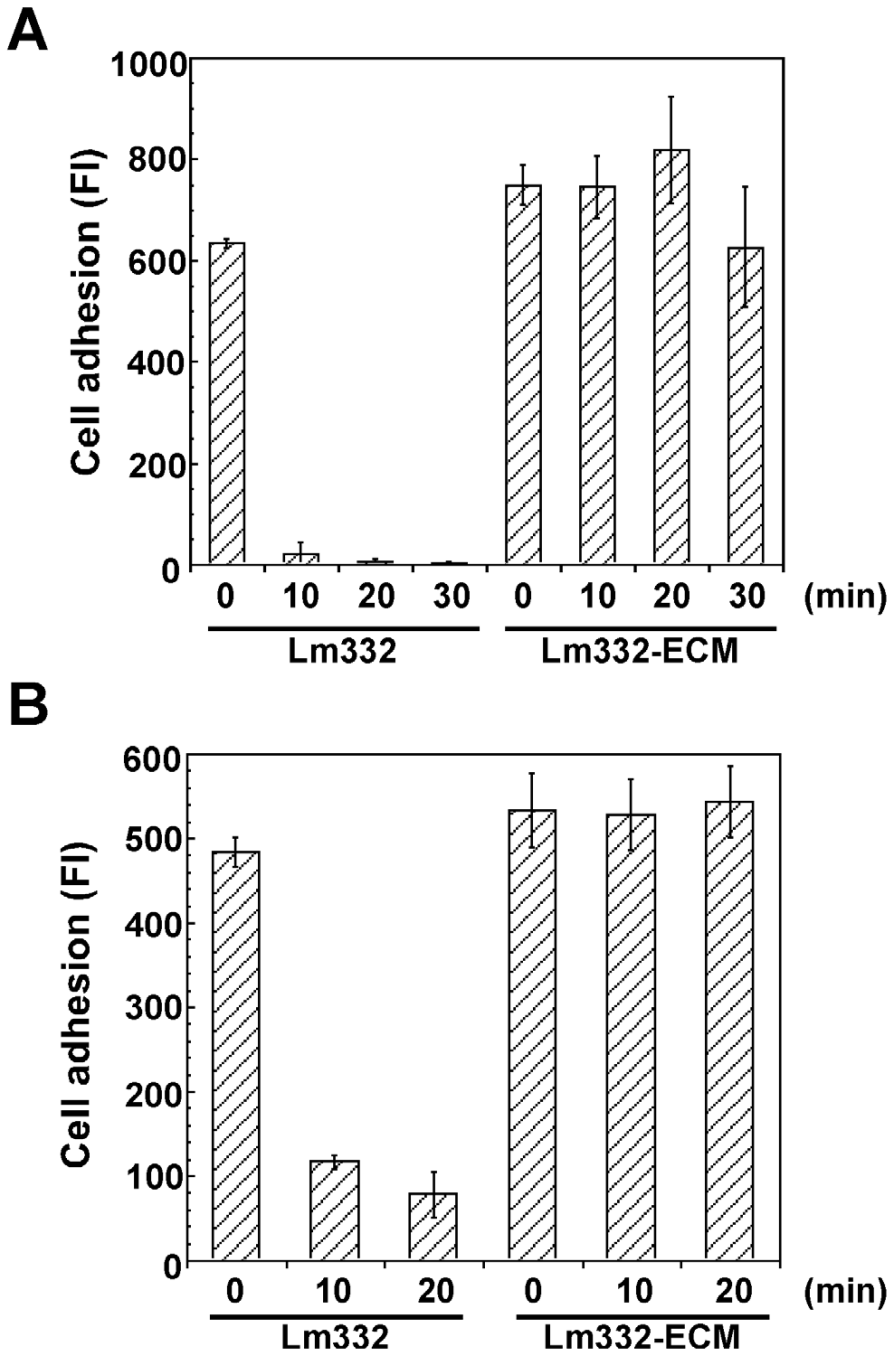
Differential detachment of NHK cells adhered to purified Lm332 and Lm332-ECM. NHK cells were inoculated onto the plates coated with 1 µg/ml purified Lm332 or deposited with Lm332-ECM and incubated for 1 h. After washing with PBS, the cells were incubated with trypsin/EDTA diluted 1∶35 in PBS (A) or with 10 mM EDTA alone (B). After incubation for the indicated lengths of time and then washing with PBS, the relative number of adherent cells was determined as described in Figure 5. Each bar indicates the mean ± S.D. of the fluorescent intensity (FI) for adherent cells in triplicate assays.

To further confirm the strong cell adhesion activity of Lm332-ECM compared to coated Lm332, we measured cell detachment by treatment with trypsin or 10 mM EDTA. After NHK cells were allowed to adhere and fully spread on Lm332-coated plates or Lm332-ECM by incubating them for 1 h, they were treated with a diluted trypsin solution for the indicated lengths of time, followed by counting the remaining attached cells. Although the cells on purified Lm332 were almost completely detached for 10 min incubation, the majority of the cells on Lm332-ECM remained attached to the plates even after 30 min (Figure 9A). Almost the same result was obtained when treated with EDTA alone: after 20 min incubation, 86% of NHK cells were detached from Lm332-coated plate but few cells from Lm332-ECM (Figure 9B). These results also indicated that NHK cells firmly adhered to Lm332-ECM compared to purified Lm332.

### Hemidesmosome Formation

**Figure 10:**
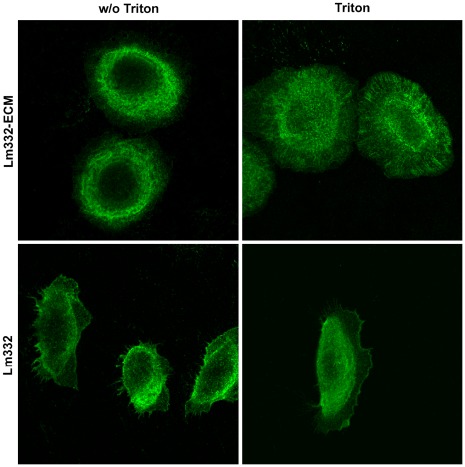
Localization of integrin ß4 in NHK cells on purified Lm332 and Lm332-ECM. Glass-bottom dishes (Asahi Techno Glass, Tokyo, Japan) were previously coated with 2.0 µg/ml Lm332 or deposited with Lm332-ECM. NHK cells were inoculated and incubated in growth media. After incubation for 5 h, the cells were washed with PBS and then fixed with 4% (w/v) paraformaldehyde in PBS for 10 min and then treated without (w/o Triton) or with (Triton) 0.5% (v/v) Triton X-100. The fixed cells were stained with an integrin ß4 antibody and an Alexa Fluor 488-labeled secondary antibody. Other experimental conditions are described in “ Materials and Methods”.

It is well known that keratinocytes produce the stable cell adhesion structure hemidesmosome by binding to Lm332 via integrin α6ß4. The hemidesmosome structure is known to remain as insoluble spots after Triton X-100 treatment [35]. To assess the hemidesmosome formation, we analyzed localization of ß4 integrin on NHK cells by immunofluorescent staining. When NHK cells were directly subjected to the immunostaining for ß4 integrin, the cells on Lm332-ECM showed strong ring-like stain with small dot signals around nucleus, whereas those on purified Lm332 were locally stained at both front and rear edges (Figure 10A, left panels). When NHK cells were immunostained after treatment with 0.5% Triton X-100, hemidesomosome-like punctuated structures of NHK cells became prominent specially at their peripheral regions on Lm332-ECM, but such peripheral staining was totally absent in the cells on purified Lm332 (Figure 10A, right panels). Based on these results, it may be concluded that NHK cells efficiently produce hemidesomosome structures containing integrin α6ß4 on Lm332 matrix but scarcely on purified Lm332.

## Discussion

In the present study, we analyzed deposition of Lm332 matrix by 7 kinds of Lm332-expressing cells including normal keratinocytes and cancer cell lines. All these kinds of cells efficiently deposited Lm332 in specific patterns onto culture plates. In a case of Lm332-HEK cells, Lm332 was an almost exclusive component in the ECM and organized into a mesh-like structure, suggesting that Lm332 was self-polymerized into the mesh structure. Furthermore, we found that the Lm332 matrix exhibited distinct activity from that of purified Lm332 protein. The former supported strong adhesion of keratinocytes but suppressed their migration as compared with the purified Lm332.

Many groups have investigated deposition and assembly of Lm332 by cultured keratinocytes [24], [29], [33]–[38]. These studies have shown that many factors including cell surface proteins, ECM proteins and intracellular signaling molecules are involved in the deposition and/or organization of laminin matrix. In this study, we could not detect any of type IV and VII collagens, perlecan and nidogen-1 in Lm332-ECM. Although the exact mechanism of laminin deposition remains to be clarified, it seems clear that secreted laminins can be deposited without support of any other ECM molecules. BM proteins such as nidogens, perlcan and type VII collagen are thought to stabilize the laminin matrix *in vivo* [29]. Cell surface receptors such as integrins, dystroglycans and sulfatides have been reported to regulate the organization and/or deposition of the laminin matrix [36], [37], [40]. In the present study, the Lm332 deposition by migrating cells was independent of Lm332-binding integrins such as integrins α3ß1, α6ß1, and α6ß4, but stationary or confluent cells seemed to interact with the self-made Lm332 matrix through these integrins, modulating the pattern of Lm332 matrix. On the other hand, sodium selenate, an effective inhibitor for the sulfation of heparan sulfates and chondroitin sulfates [41], significantly inhibited the Lm332 deposition, suggesting that heparan sulfate proteoglycans such as syndecans might play an important role in this process. It seems also possible that sulfated glycolipids on cell membrane mediate the Lm332 deposition.

Full-sized laminins such as laminin-111, laminin-211 and laminin-511 are able to self- or co-polymerize in the matrix [42], [43]. Because the LN domains of the three full-sized laminin chains are critical for the polymerization, Lm332, of which the three chains are all truncated in their short arms, has been believed to be incapable of self-polymerization or co-polymerization with other laminins [29]. In the present study, Lm332-HEK cells deposited Lm332 in a mesh-like network structure as analyzed by electron microscopy. Although ß3γ2-HEK cells deposited the ß3 and γ2 proteins, probably in a heterodimer form, such a mesh structure was not found in the ß3γ2-ECM. In addition, the deposited Lm332 matrix was not dissociated into the Lm332 heterotrimer by SDS in the absence of reducing reagent. These results strongly suggest that the Lm332 heterotrimer is able to self-polymerize in the matrix. It has been reported that the short arm of the γ2 chain [33] and the LG4-5 domain of the α3 chain [34] are important for the Lm332 deposition. By using a HEK cell line expressing Lm332 without the γ2 short arm, we have confirmed that the short arm is critical for the Lm332 deposition (unpublished data). We also found that the LG4-5 domain of the α3 chain enhances the Lm332 deposition [24] but it seems not essential [31]. The short arm of the ß3 chain does not significantly affect the Lm332 deposition but it promotes the deposition of laminin-511, suggesting its interaction with the full-length laminin chains [38]. Present analyses showed that after the deposition of Lm332, the γ2 short arm and the α3 LG4-5 domain were released by proteolytic cleavage. This suggests that they are required for the Lm332 deposition but not directly involved in the polymerization. It is expected that the interaction of the γ2 short arm with heparan sulfate proteoglycans or sulfated glycolipids plays a critical role in the efficient deposition of Lm332. Our results that Lm511 and Lm311 were not deposited on the matrix also imply the important role of the laminin γ2 short arm in the Lm332 deposition. Further studies are required to clarify how the cleaved Lm332 self-polymerizes in the matrix.

Although there were numerous studies on the biological activity of Lm332 protein, few reports compared the activities of Lm332-ECM and purified Lm332. Here we demonstrated that Lm332-ECM was clearly different from the purified, coated Lm332 substrate concerning the biological activity. It has been accepted that purified Lm332 efficiently supports cell adhesion and migration [28]. However, Lm332-ECM supported the adhesion of keratinocytes much more rapidly and strongly than the purified Lm332. The strong and stable cell adhesion to Lm332-ECM, which was evident by the resistance of keratinocytes to cell detachment treatments, lead to the suppressed cell migration. Both Lm332-ECM and ß3γ2-ECM hardly supported cell migration, but treatment of these ECMs with purified Lm332 enhanced cell migration. Even in this experiment, the Lm332-ECM coated with purified Lm332 showed suppressed cell motility activity as compared with purified Lm332 alone or ß3γ2-ECM plus Lm332. These results clearly indicate that Lm332-ECM rather inhibits cell migration.

The strong cell adhesion to Lm332-ECM obviously depends on the interaction with integrins. It has been reported that keratinocytes interact with self-deposited Lm332 through integrins α3ß1 for polarization and migration [35]. Integrin α3ß1 also functions for the proper organization of deposited Lm332 [36]. Our results indicated that integrin α3ß1 bound to Lm332-ECM in a much higher affinity than purified Lm332. In addition, experiments with neutral integrin antibodies suggested that integrin α6ß4 contributed to the cell adhesion to Lm332-ECM more greatly than that to purified Lm332. The interaction of Lm332 with the cell surface integrin α6ß4 nucleates the stable cell adhesion structure hemidesmosome in keratinocytes [8]. The present immunocytochemical staining with an anti-integrin ß4 antibody revealed numerous detergent-resistant, hemidesmosome-like structures in the cells adhered to Lm332-ECM, whereas these structures were almost absent in the cells adhered to purified Lm332. Based on all these data, it may be concluded that the polymerized Lm332 matrix strongly binds to integrins α3ß1 and α6ß4, and the latter binding promotes hemidesmosome formation, resulting in the tight and stable cell adhesion and the suppressed cell migration. Our finding is similar to the previous study that three-dimensionally organized fibronectin matrix has stronger cell adhesion activity than purified fibronectin [5].

The stable adhesion of keratinocytes to Lm332-ECM mimics the interaction of Lm332 with integrin α6ß4 in the hemidesmosome structure of basal keratinocytes *in vivo* [8]. Lm332 is known to bind to type VII collagen via the short arm of the ß3 chain, forming the anchoring filaments [44]. It is highly expected that the self-polymerization of Lm332 occurs in normal basement membranes and type VII collagen binds to the polymerized Lm332. These Lm332-dependent cell structures could support the stable anchoring of the edpidermis to the dermis in the normal skin. On the other hand, the cell motility activity of Lm332 is thought to contribute to wound repair [17] and tumor invasion [45]. We have previously reported that Lm332 stimulates cell migration in a soluble form [16]. It has been reported that matrix metalloproteinases capable of cleaving the short arm of the laminin γ2 chain are overexpressed in pathological conditions such as wound healing and cancer invasion [25], [26]. It is expected that the proteolytic cleavage of the γ2 chain prevents the polymerization and assembly of Lm332 into the basement membrane. The resultant soluble Lm332, like coated Lm332, is likely to promote the migration of normal skin cells and cancer cells in the wound healing and cancer invasion, respectively [28]. In conclusion, the present study strongly suggests that the contrasting activities of Lm332, *i.e.* the stable cell adhesion *vs*. the enhanced cell motility, are caused from the different states of Lm332, *i.e.* polymerized Lm332 matrix and nonpolymerized soluble Lm332. It is assumed that the soluble or unassembled form of Lm332 plays an important role in the elevated cell migration during the wound healing and tumor invasion.

## Materials and Methods

### Antibodies and Reagents

Mouse monoclonal antibodies against the N-terminal regions of human laminin α3A chain (LSαc3 and BG5), ß3 chain (12C) and γ2 chain (D4B5) and one against human nidogen-1 (10F) were produced in our laboratory [46]. Function-blocking anti-integrin antibodies used are anti-α2 integrin antibody (P1E6), anti-α3 integrin antibody (P1B5), anti-α5 integrin antibody (P1D6) and anti-ß1 integrin antibody (6S6) from Chemicon (Temecula, CA), and anti-α6 integrin antibody (GoH3) from PharMingen (San Diego, CA). For immunostaining, anti-laminin-γ2 chain antibody (GB3) and anti-α6 integrin (H-87) and anti-ß4 integrin (H-101) antibodies from Santa Cruz Biotechnology (Santa Cruz, CA) were also used. Other commercial antibodies to human antigens used for immunoblotting are monoclonal antibody against the laminin ß3 chain (Kalinin B1) from Transduction Laboratories (Lexington, KY), rabbit polyclonal antibody against the laminin α5 chain (LAMA A01) from Abnova (Taipei, Taiwan), anti-fibronectin antibodies (FN 12–8 and 8–12) from Takara (Tokyo, Japan), anti-type VII collagen antibody (LH7.2) from Sigma (St. Louis, MO), anti-type IV collagen antibody (H-234) from Santa Cruz, and anti-perlecan antibody (Clone 7B5) and Alexa Fluor 488-labelled secondary antibody from Invitrogen (Camarillo, CA). Human recombinant Lm332 and Lm311 were purified as described previously [30], [47]. Human recombinant Lm511 was purchased from BioLamina (Sundbyberg, Sweden).

### Cells and Transfectants

Human embryonic kidney cell line HEK293 (ATCC CRL-1573) was purchased from American Type Culture Collection and transfected with the cDNAs of the three Lm332 subunits to overexpress the wild-type Lm332 (Lm332-HEK) [30] or an α3-mutated Lm332 resistant to the proteolytic processing of the α3 chain (Lm332α3AA-HEK) [24]. Lm332-producing human cancer cell lines used were epidermoid carcinoma of the vulva (A431), epidermoid carcinoma of the cervix (CaSki), squamous adenocarcinoma of the tongue (HSC-4) and gastric adenocarcinomas (STKM-1 and MKN-45). STKM-1 was established and provided by Dr. S. Yanoma (Kanagawa Cancer Center, Yokohama, Japan) [48], and the others were obtained from Japanese Cancer Resources Bank (JCRB; Tokyo). Expression of Lm332 in these cancer cell lines were reported in our past studies [11], [32]. All these cell lines were stored in a liquid N_2_ tank in our laboratory and cultured in DMEM/F12 medium (Invitrogen, Carlsbad, CA) supplemented with 10% fetal calf serum, penicillin and streptomycin sulfate. NHK cells from neonatal foreskin were obtained from Cascade Biologics (Portland, OR), and cultured in KGM medium (Sanko-Junyaku, Tokyo, Japan), which was composed of keratinocyte basal medium, 0.1 ng/ml human EGF, 0.4% (v/v) bovine pituitary extract, 10 µg/ml insulin, 500 ng/ml hydrocortisone, 50 µg/ml gentamicin, and 50 ng/ml amphotericin B. Passages 2 and 3 were used in the described experiments.

### Preparation of CM, Deposited ECM, and Lm332-coated Plates

Unless otherwise noted, cells were grown to subconfluence in the growth medium, washed three times with PBS and then incubated in serum-free medium. Two days later, the CM was collected and added with two protease inhibitors, phenylmethylsulfonyl fluoride and *N*-ethylmaleimide. The CM was dialyzed against pure water, lyophilized and then dissolved in a 1/50 volume of PBS. To prepare deposited ECM, subconfluent cultures were incubated in the growth medium for 2 days with medium change every day. After washing twice with PBS, the cells were removed from the plates by incubating with 10 mM EDTA, and the plates were washed five times with PBS and then used for the following assays. In cases of NHK cells, the cells remaining on the plates after EDTA treatment were completely removed by further treating with 20 mM NH_4_OH for 5 min. All preparations were checked to be cell-free under a microscope. For immunoblotting analysis, the deposited ECM was dissolved in the SDS sample buffer. In some experiments, cells were directly inoculated and incubated at a high density in serum-free medium for the indicated lengths of time, and ECM and/or CM were prepared. To coat culture plates with purified Lm332 protein or CM, the plates were incubated with Lm332 or CM overnight at 4°C, briefly washed with PBS, and blocked with 1% bovine serum albumin (BSA) at room temperature for 1 h. After washing three times with PBS, the plates were used for the following assays.

### ELISA

ELISA was carried out as follows. Ninety-six-well plates coated or deposited with test substances were blocked with 2% BSA for 1 h, washed three times with PBS containing 0.1% Tween20 (PBS/Tween), and then incubated with anti-α3 (Lsαc3) or anti-γ2 chain (D4B5) antibody (diluted 1∶1000 with PBS/Tween) for 1 h at room temperature. After washing with PBS/Tween, the samples were incubated with goat anti-mouse IgG antibody coupled with biotin and then with alkaline phosphatase-conjugated avidin D. The immunosignals were visualized with p-nitrophenylphosphate and measured for absorbance at 405 nm.

### Immunofluorescence Staining of Lm332 and Integrins

To analyze the Lm332 deposition by cultured cells, Lab-Tek 8-well chamber slides (Nunc, Naperville, IL) were previously coated with 10 µg/ml bovine type I collagen (Koken, Tokyo, Japan) at 4°C overnight and washed with PBS. Cell suspension (2×10^3^ cells/0.25 ml) in serum-free medium was inoculated per well of the chamber slides and incubated for 6 h. The cultures were washed with PBS, fixed with 4% (w/v) paraformaldehyde in PBS for 10 min, and then treated with 0.5% (v/v) Triton X-100 in PBS for 15 min. The fixed cells were blocked with 2% BSA in PBS for 1 h and then reacted with the mouse anti-laminin α3 chain antibody BG5 or other antibodies for 1 h. After washing with PBS, the cultures were incubated with a mixture of FITC-conjugated goat anti-mouse IgG antibody (Vector Laboratory, Burlingham, CA) and rhodamine phalloidin (Invitrogen) for 1 h, and then washed with PBS. Fluorescence images were obtained using fluorescence microscope BZ-8000 (Keyence, Osaka, Japan) or a LSM510 confocal microscope (CarlZeiss). To immunostain Lm332 in the ECMs deposited by various types of cells, the ECMs were prepared as described above and directly subjected to the staining without the fixation. In the analysis of integrin localization, rabbit polyclonal antibodies against ß4 integrin (H-101) and α6 integrin (H-87) were used as primary antibodies.

### Cell Adhesion Assay

Cell adhesion assay with NHK cells was performed as described previously [15]. Briefly, each well of 96-well ELISA plates (Costar, Cambridge, MA) was coated with a substrate protein at indicated concentrations at 4°C overnight and then blocked with 1% BSA. Cells (2×10^4^ cells) were inoculated per well containing KGM medium, and incubated in described conditions. After non-adherent cells were removed, adherent cells were fixed and stained with Hoechst 333432. The fluorescent intensity of each well of the plates was measured using a CytoFluor 2350 fluorometer (Millipore, Bedford, MA). For inhibition assay, the cell suspension was incubated with function-blocking anti-integrin antibodies or inhibitors under the indicated conditions before inoculation.

### Cell Migration Assay

NHK cells (2.5×10^4^ cells in KGM medium) were inoculated per well of 24-well plates pre-coated with a test protein or deposited with cell-derived ECM. After pre-incubation for 1.5 h at 37°C, cell movement was monitored using a time-lapse video equipment for 5.5 h. Total length of random pass that each cell covered was measured using a video micrometer (VM-30, Olympus, Tokyo).

### SDS-PAGE and Immunoblotting

SDS-PAGE was performed on 5% gels, or 4.0–7.5% or 5.0–20% gradient gels under reducing or non-reducing conditions. Separated proteins were stained with CBB. For immunoblotting analyses, proteins resolved by SDS-PAGE were transferred to nitrocellulose membranes and detected with the ECL detection reagents (GE Healthcare, Buckinghamshire, UK).

### Integrin Binding Assay

Integrin titration assays were carried out by the method of Nishiuchi *et al*. [49]. Microtiter plates were coated with 1 µg/ml Lm332 overnight at 4°C or deposited with Lm332-ECM as described above. The wells were blocked with 1.2% BSA at room temperature for 1 h and then washed with TBS/Mn (20 mM Tris-HCl, pH7.5, 0.15 M NaCl, 1 mM CaCl_2_, 1 mM MgCl_2_, 1 mM MnCl_2_) containing 0.1% BSA and 0.02% Tween-20 (Buffer A). Serially diluted α3ß1 integrin with Buffer A was added to the plates and allowed to bind to the substrates for 3 h. For negative control, Buffer A containing 10 mM EDTA was used. The plates were washed with 25 mM HEPES (pH 7.6) containing 1 mM MnCl_2_ or 10 mM EDTA, and bound integrins were fixed with 2.5% glutaraldehyde in HEPES buffer for 10 min. The plates were washed with TBS/Mn, and the bound integrin was quantified by ELISA. Buffer A was used for the dilution of reagents and plate washing. The absorbance obtained in the presence of 10 mM EDTA was subtracted as background from each data.

### Cell Detachment Assay

NHK cells (2×10^4^ cells) were seeded into each well deposited with Lm332-ECM or coated with purified Lm332 in 96-well plates, and incubated for 1 h at 37°C. The cells were then treated with a solution of trypsin/EDTA (Cambrex Bio Science, Walkersville) diluted 1∶35 in PBS or with 10 mM EDTA/PBS for varied lengths of time. The relative number of adherent cells was determined as described in the cell adhesion assay section.

### Transmission Electron Microscopy of ECM Proteins

Confluent cultures of Lm332-HEK and ß3γ2-HEK cells were incubated on poly-L-lysine-coated cover slide glasses for 4 days, and their deposited ECMs were prepared as described above. The ECMs were then fixed with 2% glutaraldehyde and then with osmium tetraoxide. The materials were analyzed with JEOL JEM 200EX (Tokyo) at Hanaichi Electron Microscope Technology Institute (Okazaki, Japan).

## Supporting Information

Figure S1 **Immunostained patterns of Lm332 matrices deposited by normal keratinocytes (NHK) and four cancer cell lines (A431, HSC-4, STKM-1 and MKN-45).** Each kind of cells (1×10^5^ cells) were inoculated per well of Lab-Tek 8-well chamber slides in serum-free medium and incubated for 2 days. After the cells were removed by treating with 10 mM EDTA, deposited Lm332 matrices were immunostained with the anti-laminin α3 chain antibody BG5 and a FITC-conjugated secondary antibody. Other experimental conditions are described in “Materials and Methods”. Bars, 100 µm.
(TIF)

Figure S2 **Immunostaining of Lm332 matrices deposited by NHK and Lm332-HEK cells with antibodies to the laminin α3 (BG5), ß3 (12C) and γ2 (GB3) chains.** The Lm332 matrices deposited by the two types of cells during 6 h incubation were subjected to immunofluorescence staining with the three different antibodies. Other experimental conditions are the same as described in Figure S1.
(TIF)

Figure S3 **Effect of anti-integrin antibodies on Lm332 deposition by Lm332-HEK cells.** (A) Effect on cell attachment. Ninety-six-well plates were coated with 0.3 µg/ml purified Lm332 and blocked with BSA. Lm332-HEK cells suspended in serum-free medium were pretreated with non-immune mouse IgG (20 µg/ml) as a negative control or with both anti-α3 integrin (P1B5) and anti-α6 integrin (GoH3) antibodies (20 µg/ml IgG each) at 37°C for 15 min. The pretreated cells were inoculated onto the Lm332-coated plates and incubated for 1 h. After the incubation, adherent cells were determined. Each bar represents the mean ± S.D. of the fluorescent intensity (FI) for adherent cells in triplicate assays. (B) Effect on Lm332 deposition. Lm332-HEK cells treated with the control IgG (left panel) or with the anti-integrin antibodies (right panel) were inoculated on collagen-coated 8-well chamber slides and incubated for 6 h. The cultures were then stained for Lm332 with the anti-α3 chain antibody BG5 followed by a FITC-labeled secondary antibody (green) and for F-actin with rhodamine phalloidin (red). Other experimental conditions are described in Figure 2 and “Materials and Methods”.
(TIF)

Figure S4 **Quantitative assay of Lm332 deposited on culture plates by Lm332-HEK cells by ELISA and CBB staining.** Fifty µl of purified Lm332 protein (open circles) were coated at the indicated concentrations to the 96-well plates. Lm332-HEK transfectant was cultured in DMEM/F12 medium supplemented with 10% fetal calf serum, and ECM proteins (closed circles) were deposited on the plates for 3 days. The amount of Lm332 on the plates was determined by ELISA using the antibodies against the laminin α3 (A) and γ2 (B) chains. Each bar represents the mean ± S.D. for triplicate assays. The data shown are representative of at least three independent experiments performed. The Lm332 concentration on the plate was equivalent to that obtained by coating purified Lm332 at a concentration of 0.61 µg/ml or 0.56 µg/ml as analyzed for the α3 and γ2 chain, respectively. (C) A 90-mm culture dish was coated with 10 ml of 1.0 µg/ml Lm332, while another 90-mm dish was deposited with Lm332-ECM by Lm332-HEK cells as described above. The coated Lm332 and the deposited Lm332-ECM were collected by dissolving with the SDS sample buffer. A 1/3 aliquot of each extract was run on a 5–20% gradient gel and stained with CBB. The ratio of the total band intensity of Lm332-ECM to the purified Lm332 was determined to be 1.1 by the NIH image software.
(TIF)

Figure S5 **Immunoblotting analyses of Lm511, nidogen-1 and fibronectin present in CM and ECM of Lm322-HEK cells.** CM (lane 1) and ECM (lane 2) were prepared from the confluent culture of Lm332-HEK cells incubated for 3 days in serum-free medium and subjected to immunoblotting, as described in Figure 1 and “Materials and Methods”. In both cases, approximately 5% of the total sample was applied to each lane of SDS-PAGE. (A) Immunoblots for Lm511 subunits. The CM and ECM were analyzed for the laminin α3, α5, ß1 and γ1 chains. Lane 3, recombinant Lm511. (B) Immunoblots for fibronectin with the antibody FN12–8, which recognizes both human and bovine fibronectin. Lane 3, human fibronectin; lane 4, bovine fibronectin. Similar immunoblots were obtained for lanes 1–3 when human, but not bovine, fibronectin-recognizing antibody (FN 8–12) was used. (C) Nidogen-1. Immunoblotting was carried out under non-reducing conditions.
(TIF)

Figure S6 **Immunoblotting analyses of Lm332 and Lm311 in CM and ECM of MKN45 gastric carcinoma cells.** CM (lane 1) and ECM (lane 2) were prepared from the serum-free confluent culture of MKN45 cells and analyzed for the laminin α3, ß1 and γ1 chains by non-reducing immunoblotting. Lane 3, purified Lm332; lane 4, purified Lm311. The upper open arrowhead in the left panel indicates the polymerized Lm332 in the ECM (lane 2), and the two lower open arrowheads indicate the Lm332 heterotrimers with different processing (360–400 kDa). The upper major band in lane 3 seems to be an artificial Lm332-Lm332 dimer. Closed arrowheads (lanes 1 and 4 in all panels) indicate Lm311 (600 kDa).
(TIF)

Figure S7 **Video microscopy of NHK cell migration on plate coated with 1.0 µg/ml Lm332.** The cell migration was monitored by video microscopy for 5.5 h under the conditions described in Figure 6.
(MPG)

Figure S8 **Video microscopy of NHK cell migration on plate deposited with Lm332-ECM.** The cell migration was monitored by video microscopy for 5.5 h under the conditions described in Figure 6.
(MPG)

## References

[1] Adams JC, Watt FM (1993). Regulation of development and differentiation by the extracellular matrix.. Development.

[2] Giancotti FG, Ruoslahti E (1999). Integrin signaling.. Science.

[3] Kalluri R (2003). Basement membranes: structure, assembly and role in tumour angiogenesis.. Nat Rev Cancer.

[4] Colognato H, Yurchenco PD (2000). Form and function: the laminin family of heterotrimers.. Dev Dyn.

[5] Cukierman E, Pankov R, Stevens DR, Yamada KM (2001). Taking cell-matrix adhesions to the third dimension.. Science.

[6] Aumailley M, Bruckner-Tuderman L, Carter WG, Deutzmann R, Edgar D (2005). A simplified laminin nomenclature.. Matrix Biol.

[7] Baker SE, Hopkinson SB, Fitchmun M, Andreason GL, Frasier F (1996). Laminin-5 and hemidesmosomes: role of the alpha 3 chain subunit in hemidesmosome stability and assembly.. J Cell Sci 109 ( Pt.

[8] Nievers MG, Schaapveld RQ, Sonnenberg A (1999). Biology and function of hemidesmosomes.. Matrix Biol.

[9] Pulkkinen L, Christiano AM, Gerecke D, Wagman DW, Burgeson RE (1994). A homozygous nonsense mutation in the beta 3 chain gene of laminin 5 (LAMB3) in Herlitz junctional epidermolysis bullosa.. Genomics.

[10] Aberdam D, Galliano MF, Vailly J, Pulkkinen L, Bonifas J (1994). Herlitz’s junctional epidermolysis bullosa is linked to mutations in the gene (LAMC2) for the gamma 2 subunit of nicein/kalinin (laminin-5).. Nat Genet.

[11] Miyazaki K, Kikkawa Y, Nakamura A, Yasumitsu H, Umeda M (1993). A large cell-adhesive scatter factor secreted by human gastric carcinoma cells.. Proc Natl Acad Sci USA.

[12] Kikkawa Y, Umeda M, Miyazaki K (1994). Marked stimulation of cell adhesion and motility by ladsin, a laminin-like scatter factor.. J Biochem.

[13] Rousselle P, Aumailley M (1994). Kalinin is more efficient than laminin in promoting adhesion of primary keratinocytes and some other epithelial cells and has a different requirement for integrin receptors.. J Cell Biol.

[14] Hirosaki T, Mizushima H, Tsubota Y, Moriyama K, Miyazaki K (2000). Structural requirement of carboxyl-terminal globular domains of laminin alpha 3 chain for promotion of rapid cell adhesion and migration by laminin-5.. J Biol Chem.

[15] Kariya Y, Tsubota Y, Hirosaki T, Mizushima H, Puzon-McLaughlin W (2003). Differential regulation of cellular adhesion and migration by recombinant laminin-5 forms with partial deletion or mutation within the G3 domain of alpha3 chain.. J Cell Biochem.

[16] Kariya Y, Miyazaki K (2004). The basement membrane protein laminin-5 acts as a soluble cell motility factor.. Exp Cell Res.

[17] Ryan MC, Tizard R, VanDevanter DR, Carter WG (1994). Cloning of the LamA3 gene encoding the alpha 3 chain of the adhesive ligand epiligrin. Expression in wound repair.. J Biol Chem.

[18] Nguyen BP, Ryan MC, Gil SG, Carter WG (2000). Deposition of laminin 5 in epidermal wounds regulates integrin signaling and adhesion.. Curr Opin Cell Biol.

[19] Pyke C, Salo S, Ralfkiaer E, Romer J, Dano K (1995). Laminin-5 is a marker of invading cancer cells in some human carcinomas and is coexpressed with the receptor for urokinase plasminogen activator in budding cancer cells in colon adenocarcinomas.. Cancer Res.

[20] Lohi J (2001). Laminin-5 in the progression of carcinomas.. Int J Cancer.

[21] Kariya Y, Kawamura C, Tabei T, Gu J (2010). Bisecting GlcNAc residues on laminin-332 down-regulate galectin-3-dependent keratinocyte motility.. J Biol Chem.

[22] O’Toole EA, Marinkovich MP, Hoeffler WK, Furthmayr H, Woodley DT (1997). Laminin-5 inhibits human keratinocyte migration.. Exp Cell Res.

[23] Goldfinger LE, Stack MS, Jones JC (1998). Processing of laminin-5 and its functional consequences: role of plasmin and tissue-type plasminogen activator.. J Cell Biol.

[24] Tsubota Y, Yasuda C, Kariya Y, Ogawa T, Hirosaki T (2005). Regulation of biological activity and matrix assembly of laminin-5 by COOH-terminal, LG4-5 domain of alpha3 chain.. J Biol Chem.

[25] Giannelli G, Falk-Marzillier J, Schiraldi O, Stetler-Stevenson WG, Quaranta V (1997). Induction of cell migration by matrix metalloprotease-2 cleavage of laminin-5.. Science.

[26] Koshikawa N, Giannelli G, Cirulli V, Miyazaki K, Quaranta V (2000). Role of cell surface metalloprotease MT1-MMP in epithelial cell migration over laminin-5.. J Cell Biol.

[27] Ogawa T, Tsubota Y, Maeda M, Kariya Y, Miyazaki K (2004). Regulation of biological activity of laminin-5 by proteolytic processing of gamma2 chain.. J Cell Biochem.

[28] Miyazaki K (2006). Laminin-5 (laminin-332): Unique biological activity and role in tumor growth and invasion.. Cancer Sci.

[29] Hamill KJ, Kligys K, Hopkinson SB, Jones JC (2009). Laminin deposition in the extracellular matrix: a complex picture emerges.. J Cell Sci.

[30] Kariya Y, Ishida K, Tsubota Y, Nakashima Y, Hirosaki T (2002). Efficient expression system of human recombinant laminin-5.. J Biochem.

[31] Kariya Y, Yasuda C, Nakashima Y, Ishida K, Tsubota Y (2004). Characterization of laminin 5B and NH_2_-terminal proteolytic fragment of its alpha3B chain: promotion of cellular adhesion, migration, and proliferation.. J Biol Chem.

[32] Mizushima H, Miyagi Y, Kikkawa Y, Yamanaka N, Yasumitsu H (1996). Differential expression of laminin-5/ladsin subunits in human tissues and cancer cell lines and their induction by tumor promoter and growth factors.. J Biochem.

[33] Gagnoux-Palacios L, Allegra M, Spirito F, Pommeret O, Romero C (2001). The short arm of the laminin gamma2 chain plays a pivotal role in the incorporation of laminin 5 into the extracellular matrix and in cell adhesion.. J Cell Biol.

[34] Sigle RO, Gil SG, Bhattacharya M, Ryan MC, Yang TM (2004). Globular domains 4/5 of the laminin alpha3 chain mediate deposition of precursor laminin 5.. J Cell Sci.

[35] Frank DE, Carter WG (2004). Laminin 5 deposition regulates keratinocyte polarization and persistent migration.. J Cell Sci.

[36] deHart GW, Healy KE, Jones JC (2003). The role of alpha3beta1 integrin in determining the supramolecular organization of laminin-5 in the extracellular matrix of keratinocytes.. Exp Cell Res.

[37] Sehgal BU, DeBiase PJ, Matzno S, Chew TL, Claiborne JN (2006). Integrin beta4 regulates migratory behavior of keratinocytes by determining laminin-332 organization.. J Biol Chem.

[38] Nakashima Y, Kariya Y, Miyazaki K (2007). The beta3 chain short arm of laminin-332 (laminin-5) induces matrix assembly and cell adhesion activity of laminin-511 (laminin-10).. J Cell Biochem.

[39] Sonnenberg A, Calafat J, Janssen H, Daams H, van der Raaij-Helmer LM (1991). Integrin alpha 6/beta 4 complex is located in hemidesmosomes, suggesting a major role in epidermal cell-basement membrane adhesion.. J Cell Biol.

[40] Li S, Liquari P, McKee KK, Harrison D, Patel R (2005). Laminin-sulfatide binding initiates basement membrane assembly and enables receptor signaling in Schwann cells and fibroblasts.. J Cell Biol.

[41] Dietrich CP, Nader HB, Buonassisi V, Colburn P (1988). Inhibition of synthesis of heparan sulfate by selenate: possible dependence on sulfation for chain polymerization.. FASEB J.

[42] Cheng YS, Champliaud MF, Burgeson RE, Marinkovich MP, Yurchenco PD (1997). Self-assembly of laminin isoforms.. J Biol Chem.

[43] Colognato H, Yurchenco PD (1999). The laminin alpha2 expressed by dystrophic dy(2J) mice is defective in its ability to form polymers.. Curr Biol.

[44] Nakashima Y, Kariya Y, Yasuda C, Miyazaki K (2005). Regulation of cell adhesion and type VII collagen binding by the beta3 chain short arm of laminin-5: effect of its proteolytic cleavage.. J Biochem.

[45] Kariya Y, Kariya Y, Gu J (2009). Roles of laminin-332 and alpha6beta4 integrin in tumor progression.. Mini Rev Med Chem.

[46] Kariya Y, Mori T, Yasuda C, Watanabe N, Kaneko Y (2008). Localization of laminin alpha3B chain in vascular and epithelial basement membranes of normal human tissues and its down-regulation in skin cancers.. J Mol Histol.

[47] Mori T, Kariya Y, Ogawa T, Higashi S, Miyazaki K (2010). Laminin-3B11, a novel vascular-type laminin capable of inducing prominent lamellipodial protrusions in microvascular endothelial cells.. J Biol Chem.

[48] Arimura A, Nakamura Y, Shimizu A, Harada M, Yanoma S (1991). Establishment and characterization of a CA19-9 producing human gastric cancer cell line, STKM-1.. Hum Cell.

[49] Nishiuchi R, Murayama O, Fujiwara H, Gu J, Kawakami T (2003). Characterization of the ligand-binding specificities of integrin alpha3beta1 and alpha6beta1 using a panel of purified laminin isoforms containing distinct alpha chains.. J Biochem.

