# Supplementary figures and images for: Polymerized Laminin-332 Matrix Supports Rapid and Tight Adhesion of Keratinocytes, Suppressing Cell Migration
Source: PLoS One. 2012 May 1;7(5):e35546. doi: 10.1371/journal.pone.0035546 (PMC3341393; doi:10.1371/journal.pone.0035546)

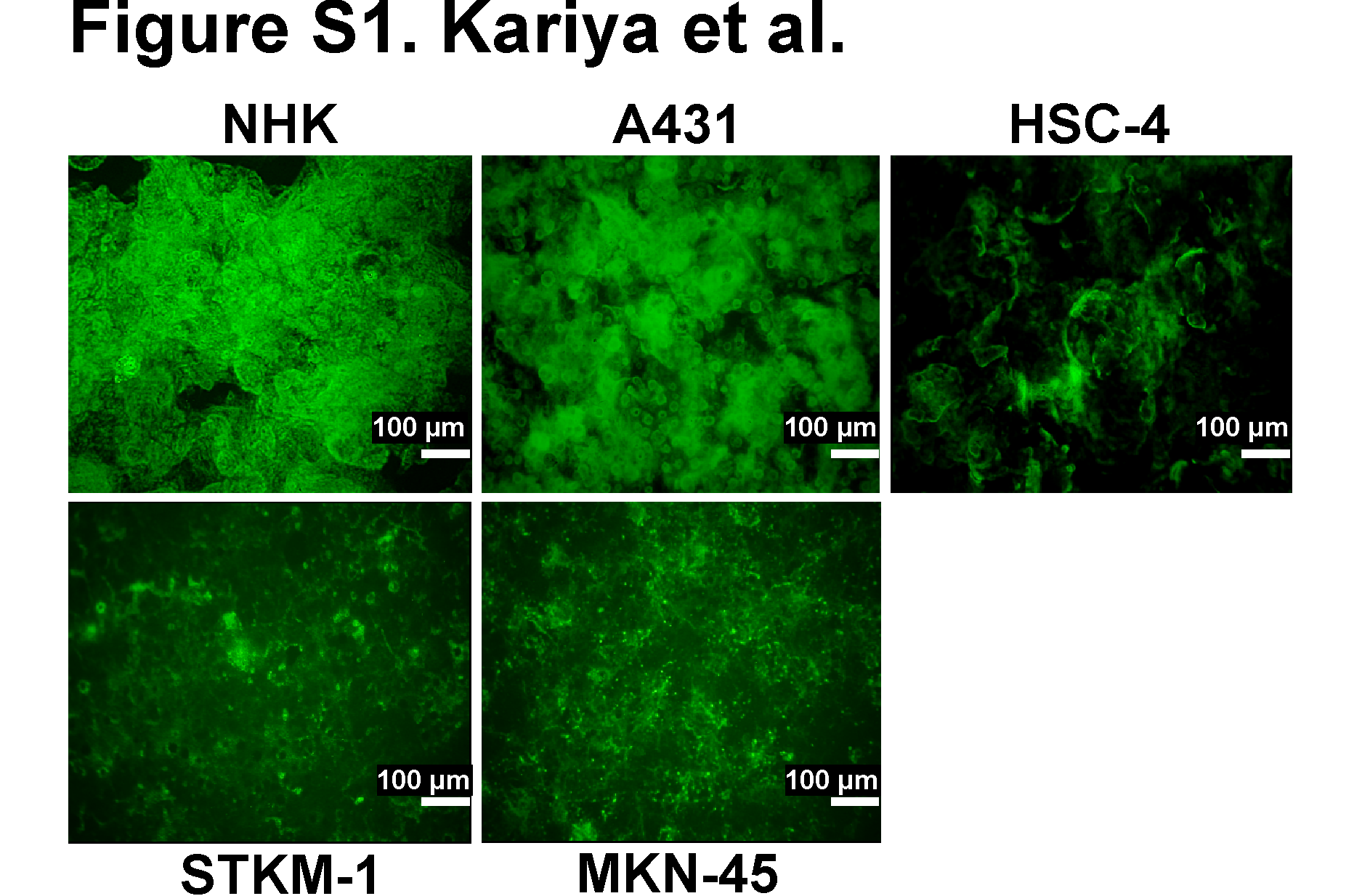

Supplement: Figure S1 — Immunostained patterns of Lm332 matrices deposited by normal keratinocytes (NHK) and four cancer cell lines (A431, HSC-4, STKM-1 and MKN-45). Each kind of cells (1×10^5^ cells) were inoculated per well of Lab-Tek 8-well chamber slides in serum-free medium and incubated for 2 days. After the cells were removed by treating with 10 mM EDTA, deposited Lm332 matrices were immunostained with the anti-laminin α3 chain antibody BG5 and a FITC-conjugated secondary antibody. Other experimental conditions are described in “Materials and Methods”. Bars, 100 µm. (TIF) [file pone.0035546.s001.tif]

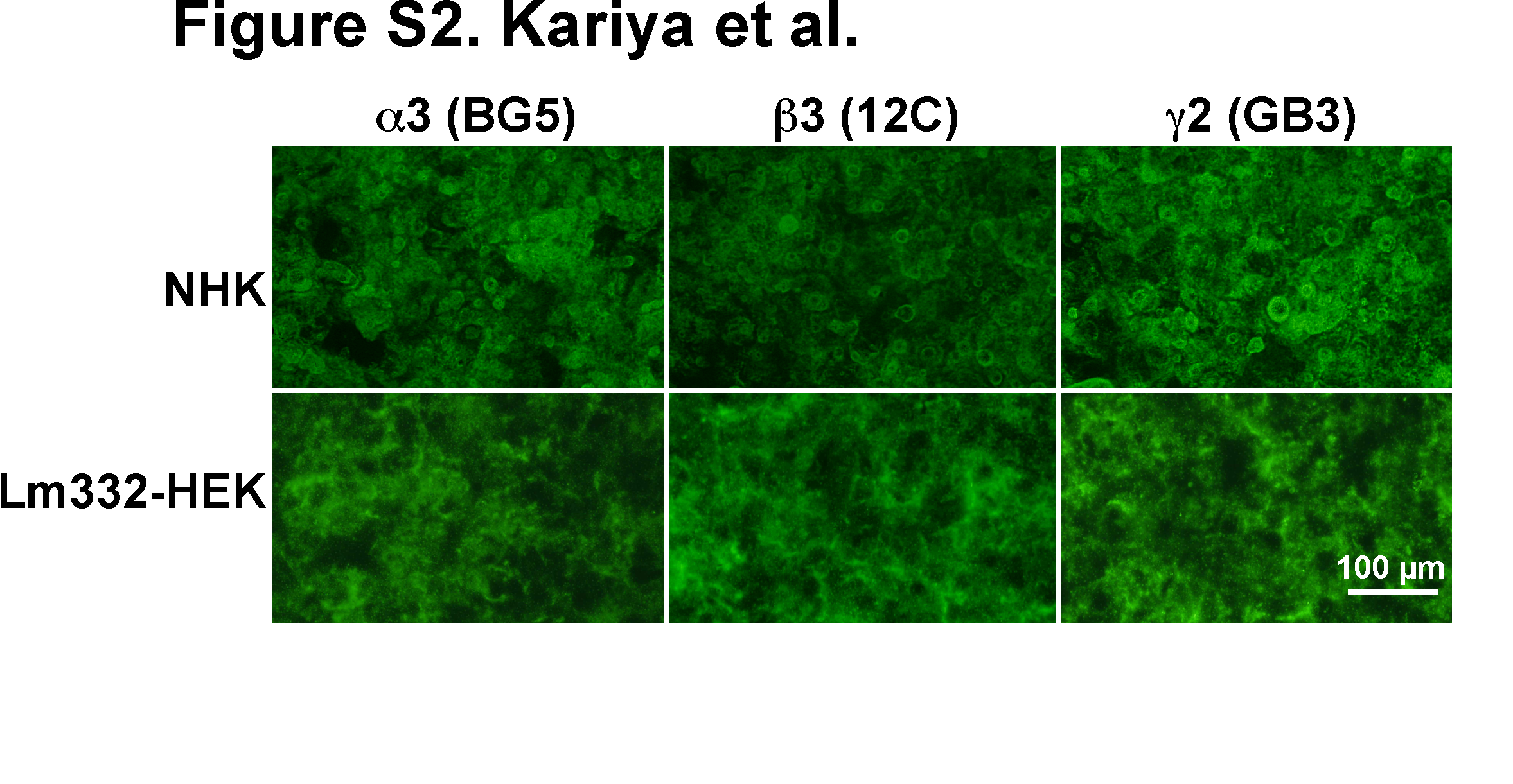

Supplement: Figure S2 — Immunostaining of Lm332 matrices deposited by NHK and Lm332-HEK cells with antibodies to the laminin α3 (BG5), ß3 (12C) and γ2 (GB3) chains. The Lm332 matrices deposited by the two types of cells during 6 h incubation were subjected to immunofluorescence staining with the three different antibodies. Other experimental conditions are the same as described in Figure S1. (TIF) [file pone.0035546.s002.tif]

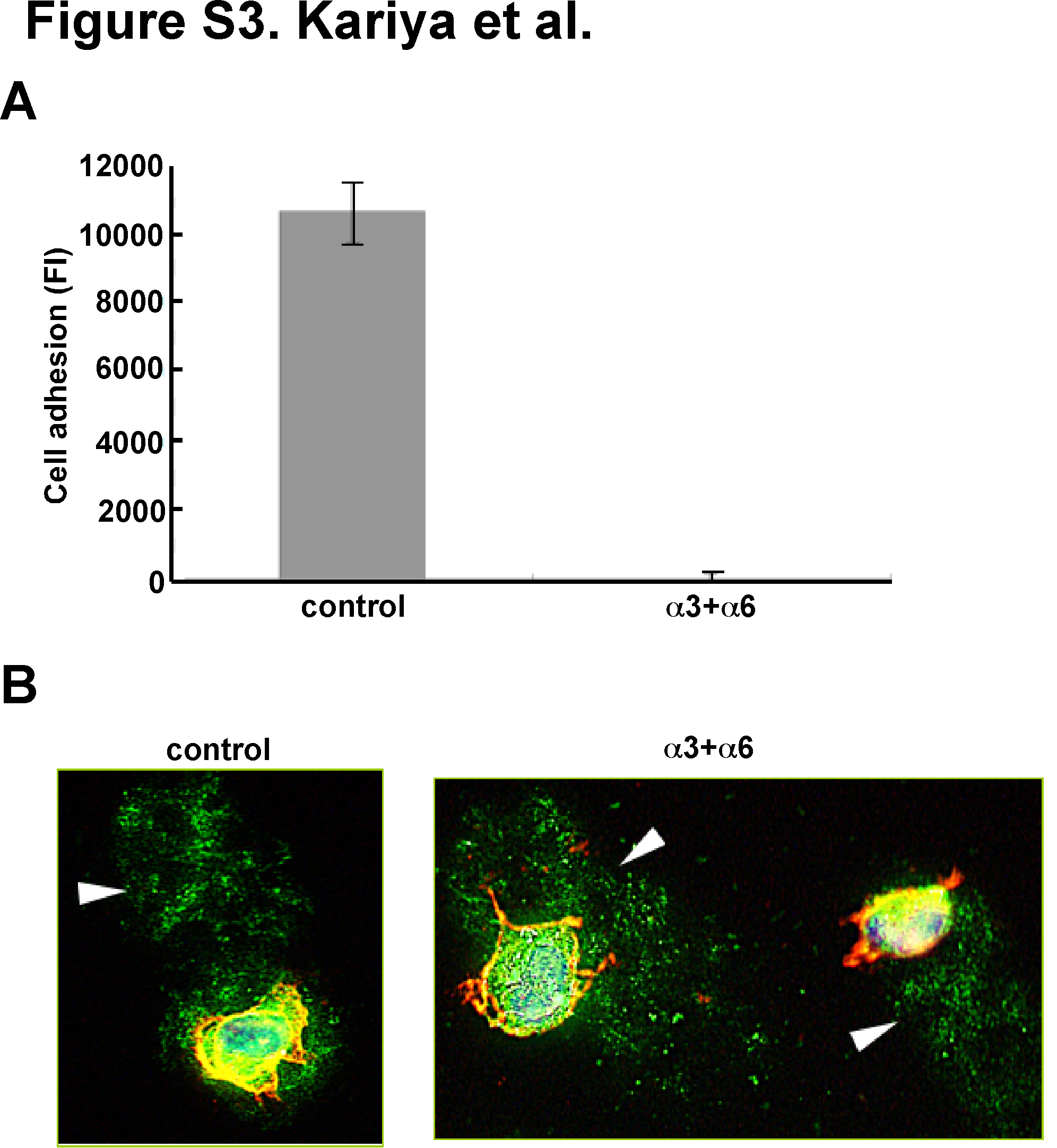

Supplement: Figure S3 — Effect of anti-integrin antibodies on Lm332 deposition by Lm332-HEK cells. (A) Effect on cell attachment. Ninety-six-well plates were coated with 0.3 µg/ml purified Lm332 and blocked with BSA. Lm332-HEK cells suspended in serum-free medium were pretreated with non-immune mouse IgG (20 µg/ml) as a negative control or with both anti-α3 integrin (P1B5) and anti-α6 integrin (GoH3) antibodies (20 µg/ml IgG each) at 37°C for 15 min. The pretreated cells were inoculated onto the Lm332-coated plates and incubated for 1 h. After the incubation, adherent cells were determined. Each bar represents the mean ± S.D. of the fluorescent intensity (FI) for adherent cells in triplicate assays. (B) Effect on Lm332 deposition. Lm332-HEK cells treated with the control IgG (left panel) or with the anti-integrin antibodies (right panel) were inoculated on collagen-coated 8-well chamber slides and incubated for 6 h. The cultures were then stained for Lm332 with the anti-α3 chain antibody BG5 followed by a FITC-labeled secondary antibody (green) and for F-actin with rhodamine phalloidin (red). Other experimental conditions are described in Figure 2 and “Materials and Methods”. (TIF) [file pone.0035546.s003.tif]

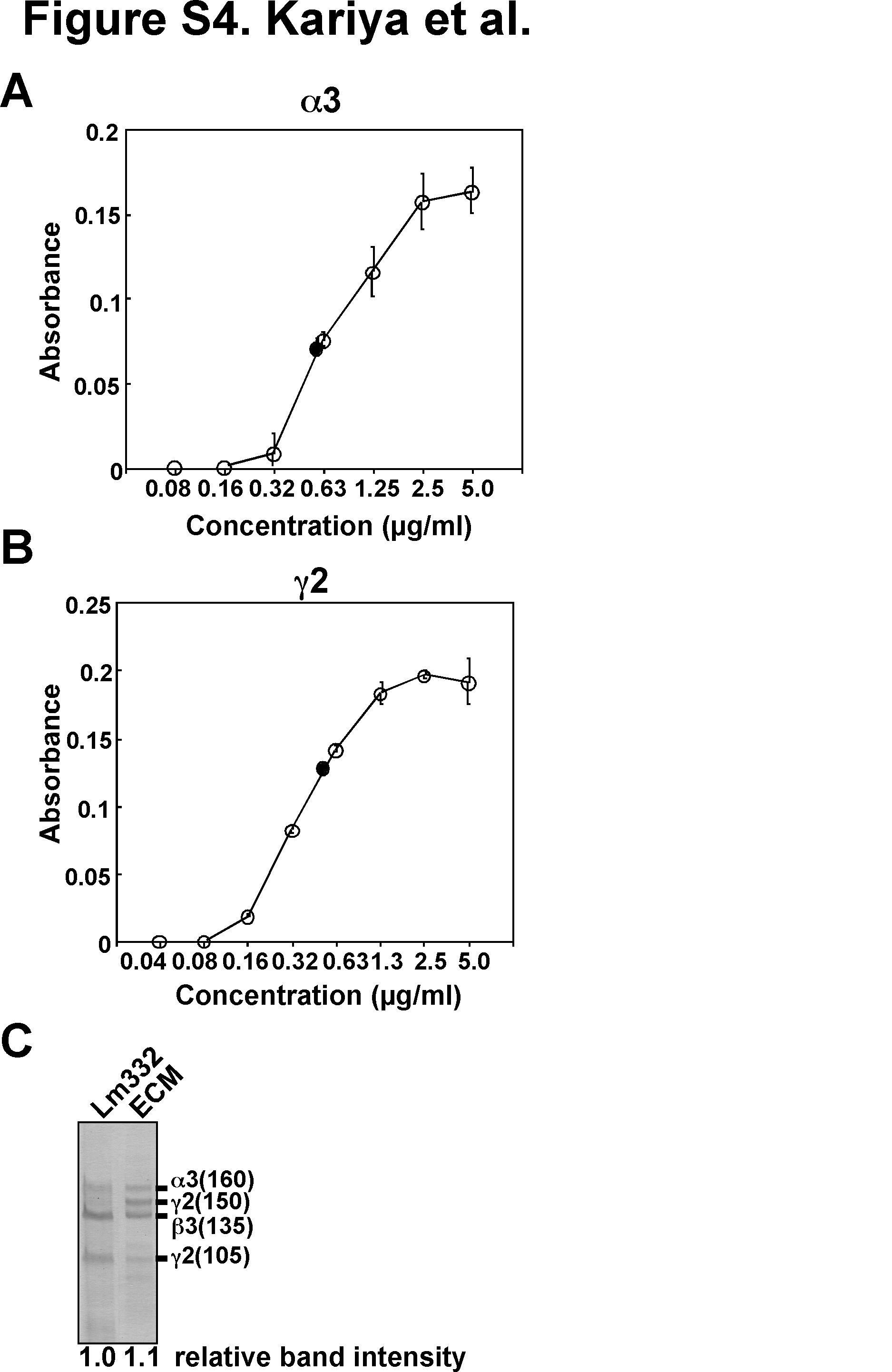

Supplement: Figure S4 — Quantitative assay of Lm332 deposited on culture plates by Lm332-HEK cells by ELISA and CBB staining. Fifty µl of purified Lm332 protein (open circles) were coated at the indicated concentrations to the 96-well plates. Lm332-HEK transfectant was cultured in DMEM/F12 medium supplemented with 10% fetal calf serum, and ECM proteins (closed circles) were deposited on the plates for 3 days. The amount of Lm332 on the plates was determined by ELISA using the antibodies against the laminin α3 (A) and γ2 (B) chains. Each bar represents the mean ± S.D. for triplicate assays. The data shown are representative of at least three independent experiments performed. The Lm332 concentration on the plate was equivalent to that obtained by coating purified Lm332 at a concentration of 0.61 µg/ml or 0.56 µg/ml as analyzed for the α3 and γ2 chain, respectively. (C) A 90-mm culture dish was coated with 10 ml of 1.0 µg/ml Lm332, while another 90-mm dish was deposited with Lm332-ECM by Lm332-HEK cells as described above. The coated Lm332 and the deposited Lm332-ECM were collected by dissolving with the SDS sample buffer. A 1/3 aliquot of each extract was run on a 5–20% gradient gel and stained with CBB. The ratio of the total band intensity of Lm332-ECM to the purified Lm332 was determined to be 1.1 by the NIH image software. (TIF) [file pone.0035546.s004.tif]

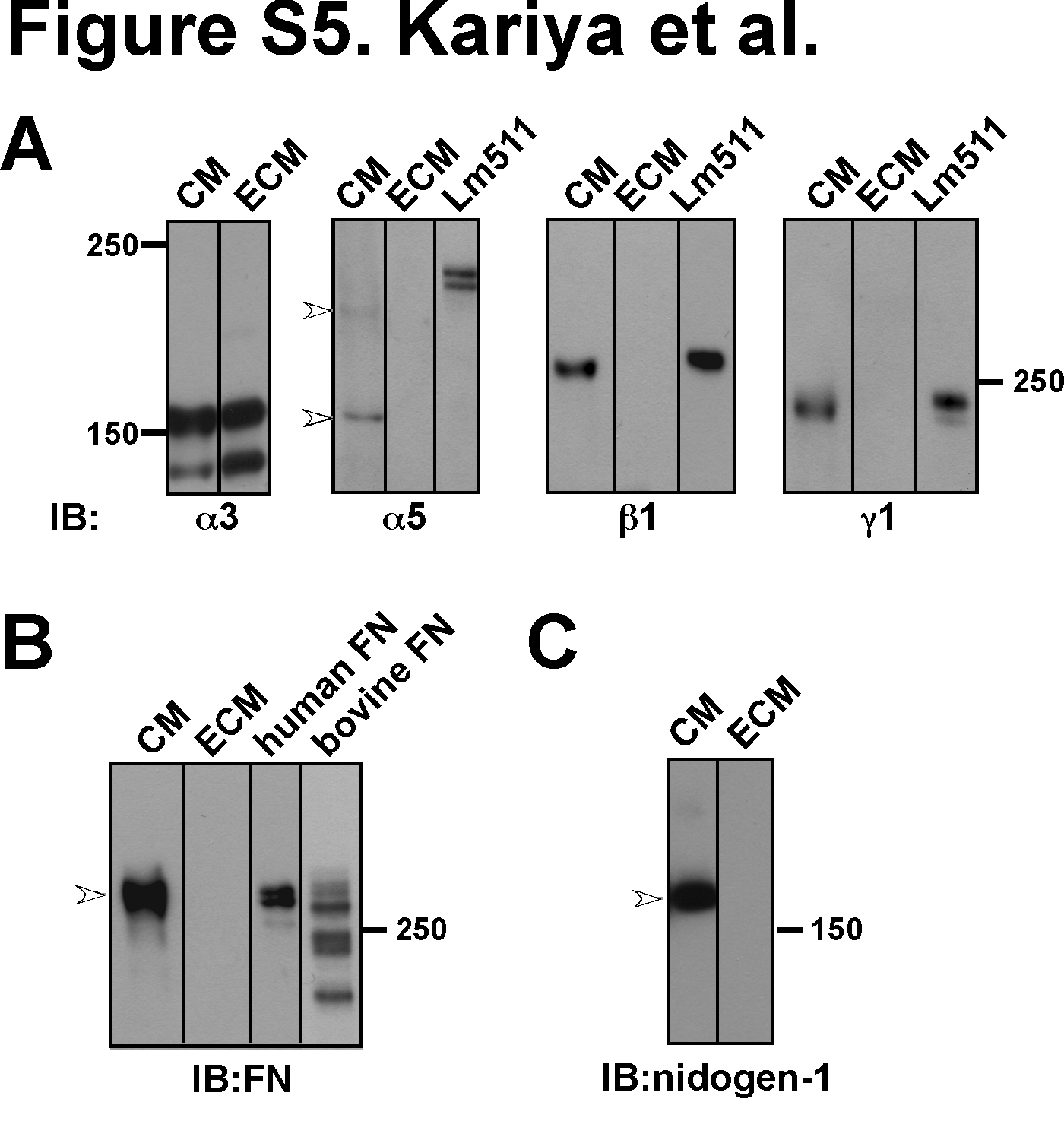

Supplement: Figure S5 — Immunoblotting analyses of Lm511, nidogen-1 and fibronectin present in CM and ECM of Lm322-HEK cells. CM (lane 1) and ECM (lane 2) were prepared from the confluent culture of Lm332-HEK cells incubated for 3 days in serum-free medium and subjected to immunoblotting, as described in Figure 1 and “Materials and Methods”. In both cases, approximately 5% of the total sample was applied to each lane of SDS-PAGE. (A) Immunoblots for Lm511 subunits. The CM and ECM were analyzed for the laminin α3, α5, ß1 and γ1 chains. Lane 3, recombinant Lm511. (B) Immunoblots for fibronectin with the antibody FN12–8, which recognizes both human and bovine fibronectin. Lane 3, human fibronectin; lane 4, bovine fibronectin. Similar immunoblots were obtained for lanes 1–3 when human, but not bovine, fibronectin-recognizing antibody (FN 8–12) was used. (C) Nidogen-1. Immunoblotting was carried out under non-reducing conditions. (TIF) [file pone.0035546.s005.tif]

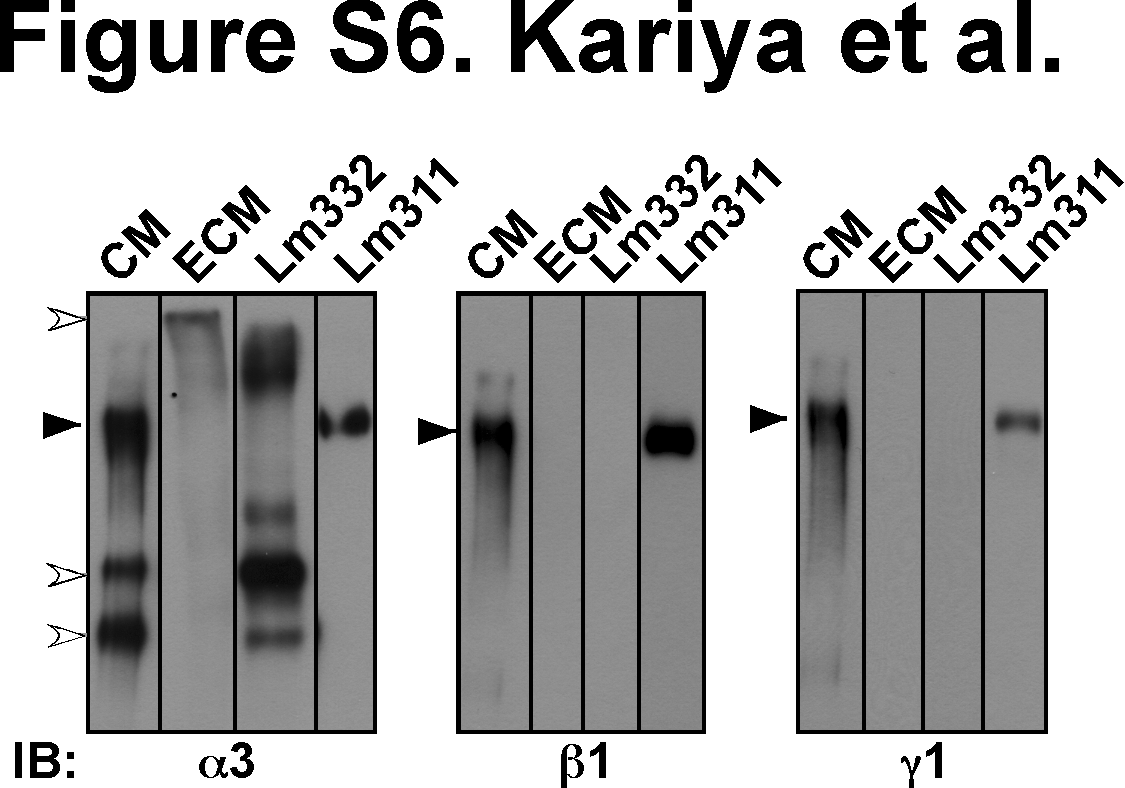

Supplement: Figure S6 — Immunoblotting analyses of Lm332 and Lm311 in CM and ECM of MKN45 gastric carcinoma cells. CM (lane 1) and ECM (lane 2) were prepared from the serum-free confluent culture of MKN45 cells and analyzed for the laminin α3, ß1 and γ1 chains by non-reducing immunoblotting. Lane 3, purified Lm332; lane 4, purified Lm311. The upper open arrowhead in the left panel indicates the polymerized Lm332 in the ECM (lane 2), and the two lower open arrowheads indicate the Lm332 heterotrimers with different processing (360–400 kDa). The upper major band in lane 3 seems to be an artificial Lm332-Lm332 dimer. Closed arrowheads (lanes 1 and 4 in all panels) indicate Lm311 (600 kDa). (TIF) [file pone.0035546.s006.tif]
